# Gut microbiota-associated immunomodulation contributes to the protective effects of fluvastatin against endometriosis in a mouse model, accompanied by increased *Akkermansia muciniphila* abundance

**DOI:** 10.3389/fmicb.2026.1762444

**Published:** 2026-05-14

**Authors:** Huilin Yang, Shuai Liu, Xia Chen, Chenxi Yin, Longxia Xiao, Wenmin Xu, Shuaijun Lv, Li Xie, Chunhua Yin

**Affiliations:** 1 Department of Gynecology and Obstetrics, First Affiliated Hospital of Nanchang University, Nanchang, Jiangxi, China; 2 Department of Neurosurgery, First Affiliated Hospital of Nanchang University, Nanchang, Jiangxi, China; 3 Department of Obstetrics and Gynecology, The First Affiliated Hospital of Ningbo University, Ningbo, China; 4 Ningbo Key Laboratory of Human Microbiome and Precision Medicine, Central Laboratory of the Medical Research Center, The First Affiliated Hospital of Ningbo University, Ningbo, China; 5 School of Medicine, Xi'an Jiaotong University, Xi'an, Shaanxi, China

**Keywords:** *Akkermansia muciniphila*, endometriosis, fluvastatin, gut microbiota, immune regulation

## Abstract

**Background:**

Endometriosis (EMs) is a chronic inflammatory disease characterized by tumor-like growth behavior and limited therapeutic options. Increasing evidence suggests that gut microbiota may contribute to EMs progression by promoting chronic inflammation and immune dysregulation. Fluvastatin, a lipid-lowering agent, exhibits anti-inflammatory, anti-tumor, and immunomodulatory effects and has also been reported to influence microbial homeostasis. However, the relationship among fluvastatin treatment, gut microbiota, and EMs progression remains unclear. This study aimed to investigate this relationship.

**Materials and methods:**

A mouse model of EMs was established by autologous uterine tissue transplantation, followed by oral fluvastatin administration for 3 weeks. Lesion growth, inflammatory responses, and immune characteristics were evaluated by histology, quantitative PCR, flow cytometry, immunofluorescence, and immunohistochemistry. Gut microbiota involvement was assessed using antibiotic-mediated microbiota depletion and fecal microbiota transplantation (FMT). Microbial composition was analyzed by metagenomic sequencing. The role of *Akkermansia muciniphila* was evaluated by direct oral supplementation.

**Results:**

Fluvastatin significantly reduced the volume and mass of ectopic lesions and decreased the mRNA expression of pro-inflammatory cytokines. It was also associated with changes in macrophage polarization-related markers and reduced abnormal activation of splenic immune cells. Antibiotic-induced gut microbiota depletion attenuated the protective effects associated with fluvastatin treatment, whereas FMT from fluvastatin-treated mice partially transferred similar protective changes. Metagenomic analysis revealed that fluvastatin reshaped gut microbiota composition and increased the abundance of *Akkermansia muciniphila*. Moreover, oral supplementation with *Akkermansia muciniphila* attenuated EMs progression and was associated with anti-inflammatory and immune-related changes similar to those observed after fluvastatin treatment.

**Conclusion:**

These findings suggest that the protective effects associated with fluvastatin treatment are accompanied by changes in gut microbiota composition, including increased abundance of *Akkermansia muciniphila*. Gut microbiota may contribute to the beneficial effects of fluvastatin in EMs. These results support the potential value of microbiota-informed therapeutic strategies for EMs.

## 1 Introduction

Endometriosis (EMs) is a chronic inflammatory gynecological disorder characterized by the presence of endometrial-like tissue outside the uterus. It affects approximately 190 million women of reproductive age worldwide, accounting for 5–10% of this population, and represents a leading cause of chronic pelvic pain and infertility, severely impairing quality of life (Taylor et al., 2021, Horne and Missmer, 2022). Although EMs is considered a benign disease, it exhibits several tumor-like features, including local invasion, recurrence, and occasional malignant transformation, which pose significant challenges for early diagnosis and effective treatment (Pearce et al., 2012; Tang et al., 2024).

Despite extensive research, the pathogenesis of EMs remains incompletely understood. Current theories involve retrograde menstruation, estrogen-dependent microenvironment, chronic inflammation, immune dysregulation, aberrant angiogenesis, and genetic susceptibility (Zondervan et al., 2018; Zondervan et al., 2020). Hormonal therapy and surgical intervention remain the primary treatment strategies; however, both approaches have notable limitations. Hormonal treatments are often associated with adverse effects and limited long-term efficacy (Horne and Missmer, 2022), while surgical resection carries a risk of complications and a high recurrence rate (Zondervan et al., 2020; Vercellini et al., 2009; Leborne et al., 2022), underscoring the urgent need for alternative therapeutic strategies.

Accumulating evidence suggests that the gut microbiota (GM) may contribute to the initiation and progression of EMs (Tang et al., 2024; Qi et al., 2021). As the largest microbial ecosystem in the human body, GM is essential for regulating inflammatory responses and maintaining immune homeostasis (Simpson et al., 2023; Ansaldo et al., 2021; Yang and Cong, 2021). Both clinical and experimental studies have consistently reported gut dysbiosis in EMs, characterized by increased abundance of potentially pathogenic bacteria, such as Proteobacteria and Enterobacteriaceae, and reduced levels of beneficial microbes, including *Akkermansia muciniphila* and *Lactobacillus* (Kovács et al., 2021; Leonardi et al., 2020; Parpex et al., 2024). This microbial imbalance has been associated with elevated IL-17 signaling, increased production of pro-inflammatory cytokines (e.g., VEGF, TNF-*α*, and IL-1β), and impaired immune surveillance through dysregulation of CD8^+^ T cells and macrophages (Zhang et al., 2005; Giudice, 2010; Ata et al., 2019; Anderson, 2019; Roy and Trinchieri, 2017). Moreover, animal studies have demonstrated that depletion of gut microbiota suppresses ectopic lesion formation, whereas transplantation of microbiota from EMs donors accelerates disease progression (Chadchan et al., 2019; Chadchan et al., 2023; Talwar et al., 2025). These findings suggest that disrupted GM may contribute to EMs pathogenesis and may represent a promising therapeutic target.

In parallel with the growing interest in the GM–EMs axis, increasing attention has been directed toward the pleiotropic effects of statins. Beyond their lipid-lowering activity through inhibition of HMG-CoA reductase, statins have been shown to exert anti-inflammatory, anti-tumor, and immunomodulatory effects and to modulate gut microbiota composition and diversity (Libby, 2020; Vieira-Silva et al., 2020; She et al., 2024). Among them, fluvastatin (Flu) is well recognized for its potent anti-inflammatory and immunoregulatory properties (Muffova et al., 2025; Lörchner et al., 2023; Assié et al., 2025; Sławińska-Brych et al., 2014; Nyambuya et al., 2021; Chen et al., 2025). Although several reviews have proposed the therapeutic potential of statins in EMs (Laschke and Menger, 2012; Chen et al., 2019), experimental evidence remains limited (Qin et al., 2024). Importantly, the relationship among Flu treatment, gut microbiota, and EMs progression remains unclear.

In this study, we used a mouse model of EMs to investigate whether the protective effects associated with Flu treatment were accompanied by changes in gut microbiota composition and immune regulation. Using a surgically induced mouse model of EMs in combination with broad-spectrum antibiotic-mediated microbiota depletion, fecal microbiota transplantation (FMT), and metagenomic sequencing, we explored the interactions among Flu, gut microbiota, and EMs progression, with particular attention to *Akkermansia muciniphila* as a candidate functional bacterium.

## 2 Materials and methods

### 2.1 Animals and ethical approval

Female C57BL/6 J mice (6–8 weeks old, 18–21 g) were purchased from SPF Biotechnology Co., Ltd. (Beijing, China). Animals were housed under controlled conditions (22 ± 1 °C, 60 ± 10% humidity) with a 12:12 h light–dark cycle and free access to food and water. Five mice were maintained per cage. All procedures complied with the Guidelines for the Care and Use of Laboratory Animals and were approved by the Ethics Committee of the First Affiliated Hospital of Ningbo University.

### 2.2 Experimental design

After 1 week of acclimation, mice were subjected to EMs model induction according to the experimental protocol. After recovery from surgery and confirmation of stable health status, animals were randomly assigned to treatment groups. Group allocation was performed independently of body weight, appearance, or postoperative condition. The experimental groups included an EMs group, an EMs + Flu group, antibiotic cocktail (ABX) groups, FMT groups, and *A. muciniphila* treatment groups. Details of each intervention are described in the following sections. For outcome assessment, investigators performing lesion measurement, histological evaluation, and flow cytometry analysis were blinded to group allocation. Samples were coded prior to analysis, and group identities were revealed after data collection was completed.

### 2.3 EMs model induction

EMs was induced using an autologous uterine tissue transplantation method as previously described (Vernon and Wilson, 1985). Mice were intramuscularly injected with 3 μg estradiol benzoate (MedChemexpress, Cat# HY-B1192, USA) to promote endometrial proliferation (Zhuang et al., 2025). On the final day of estrogen injection, mice were anesthetized by intraperitoneal injection of a mixture of ketamine (100 mg/kg) and xylazine (10 mg/kg) in 0.9% saline, and surgical induction of EMs was then performed. At the end of the experiment, mice were humanely euthanized by cervical dislocation under deep anesthesia.

### 2.4 Flu administration

On the day following EMs induction, mice were randomly assigned to the EMs or EMs + Flu group. Flu [50 mg/kg (Yu et al., 2021); Aladdin] was dissolved in phosphate-buffered saline (PBS) and administered by daily oral gavage (200 μL) for 21 days. The EMs group received an equal volume of PBS.

### 2.5 Gut microbiota depletion

GM was depleted using an ABX consisting of vancomycin (100 mg/kg, Aladdin), ampicillin sodium, metronidazole, and neomycin sulfate (each 200 mg/kg; Macklin) (Chen et al., 2023; Li et al., 2025). ABX was administered for 3 consecutive days before EMs induction. After model establishment, mice received Flu or PBS daily, while ABX groups continued to receive antibiotics every other day until the end of the experiment. In addition, to evaluate whether antibiotic treatment alone alters baseline immune status, an additional short-term experiment was performed in normal mice. Animals were randomly assigned to Ctrl or Ctrl+ABX groups (*n* = 6 per group). The Ctrl+ABX group received the same antibiotic cocktail regimen for 10 consecutive days by oral gavage, and splenic immune cells were subsequently analyzed by flow cytometry as described below.

### 2.6 FMT

Recipient mice were pretreated with the ABX cocktail for 3 days to deplete endogenous GM. Fresh feces were collected at the endpoint of the fluvastatin experiment from donor mice in the EMs and EMs + Flu groups (*n* = 10 per group). To minimize inter-individual variability, fecal samples from all donor mice within each group were pooled prior to suspension preparation. Feces were homogenized in PBS (0.125 g/mL), vortexed, and centrifuged (1,000 rpm; 1 min). Supernatants were mixed with sterile glycerol to a final concentration of 20%, aliquoted, and stored at −80 °C (Chen et al., 2022; Zeng et al., 2025). At the same time point, fecal samples from individual donor mice were collected separately for metagenomic sequencing to characterize donor microbial composition. After EMs induction, FMT recipients received 200 μL of fecal suspension or PBS daily by gavage until the experiment concluded.

### 2.7 *A. muciniphila* cultivation and administration

*A. muciniphila* (ATCC BAA-835) was grown anaerobically at 37 °C in BHI broth (Hope bio, Qingdao, China) supplemented with 0.3% mucin (Yuanye Bio-Technology, China) (Wang et al., 2025b; Yoon et al., 2021). Bacteria were passaged every 2–3 days. *A. muciniphila* was administered by oral gavage at 1.5 × 10^9^ colony-forming units (CFU) per mouse in 200 μL of PBS (Grander et al., 2018), starting the day after EMs induction and continuing daily throughout the modeling period. Mice in the EMs control group received an equal volume (200 μL) of sterile PBS by oral gavage following the same schedule.

### 2.8 Quantitative real-time PCR

Total RNA was extracted from tissue samples using an RNA extraction kit (Vazyme Biotech, Cat# RC112-01, China). cDNA was synthesized using the ReverTra Ace RT Kit (Toyobo, Cat# FSQ-101, Japan). Quantitative PCR was performed on a Cobas z 480 system (Roche, China) using SYBR Green PCR Master Mix (Toyobo, Cat# QPK-201B, Japan). 18S rRNA served as the internal reference. Relative mRNA expression was calculated using the 2^−ΔΔCt method. Primer sequences are listed in Table 1.

**Table 1:** Primers used in qPCR analysis.

Primer	Forward (5′–3′)	Reverse (5′–3′)
18sRNA	AGTCCCTGCCCTTTGTACACA	CGATCCGAGGGCCTCACTA
IL-1β	GAAATGCCACCTTTTGACAGTG	TGGATGCTCTCATCAGGACAG
TNF-α	CAGGCGGTGCCTATGTCTC	CGATCACCCCGAAGTTCAGTAG
iNOS	ACATCGACCCGTCCACAGTAT	CAGAGGGGTAGGCTTGTCTC
CD86	TTGTGTGTGTTCTGGAAACGGAG	AACTTAGAGGCTGTGTTGCTGGG
CD206	TCTTTGCCTTTCCCAGTCTCC	TGACACCCAGCGGAATTTC
Arg1	GAACACGGCAGTGGCTTTAAC	TGCTTAGCTCTGTCTGCTTTGC
16S	ACTCCTACGGGAGGCAGCAG	ATTACCGCGGCTGCTGG
*A. muc*	CAGCACGTGAAGGTGGGGAC	CCTTGCGGTTGGCTTCAGAT

### 2.9 Flow cytometry

Spleens were minced and filtered through 70 μm strainers. Red blood cells were lysed with 1× ACK buffer (Elabscience, Cat# E-CK-A105, China). After washing, cells were stained at 4 °C in sequence: Fc receptor blocking (30 min), surface marker staining (60 min), and viability dye staining (30 min), with PBS washes between steps. Cells were then resuspended in 400 μL PBS, filtered through 40 μm strainers, and analyzed using a BD FACSMelody™ cytometer (USA). Antibodies included CD45-PE-Cy7, CD3-PerCP-Cy5.5, CD4-FITC, CD8-APC, CD11b-FITC, F4/80-APC, CD19-FITC, NK1.1-APC, Gr-1-PE, Ly6G-APC, and Ly6C-PerCP-Cy5.5 (Invitrogen, USA).

### 2.10 Histopathology

Ectopic endometrial lesions and liver tissues were collected, preserved in 4% paraformaldehyde, dehydrated using ethanol, embedded in paraffin, and then sliced into 5 μm thick sections. The sections were rehydrated, stained with hematoxylin and eosin (H&E), then dehydrated and sealed. Tissue morphology was examined under an optical microscope.

### 2.11 Immunofluorescence staining

Paraffin-embedded tissue sections were deparaffinized, rehydrated, washed with PBS, and subjected to heat-induced antigen retrieval. After blocking with blocking solution (Cat# G2052, Servicebio, Wuhan, China) for 30 min at room temperature, the sections were incubated overnight at 4 °C with primary antibodies against CD86 (Cat# DF6332, Affinity, Jiangsu, China) or CD206 (Cat# DF4149, Affinity, Jiangsu, China). After washing with PBS, the sections were incubated for 2 h at room temperature in the dark with the corresponding fluorescent secondary antibodies, including Alexa Fluor 647-conjugated Goat-anti-Rabbit IgG (Cat# G-RB647, Oasisbiofarm, Zhejiang, China) and Alexa Fluor 488-conjugated secondary antibody (Cat# A-11008, Invitrogen, USA). Nuclei were counterstained with DAPI solution (Cat# C1006, Beyotime, Shanghai, China). The sections were mounted with SlowFade Glass antifade mounting medium (Cat# S36917, Invitrogen, USA) and imaged using a laser scanning confocal microscope (FV4000, Olympus).

### 2.12 Immunohistochemistry

Paraffin-embedded tissue sections were baked at 60 °C for 40 min, deparaffinized in xylene, and rehydrated through graded ethanol. Antigen retrieval was performed in citrate buffer (Cat# C1032, Solarbio, Beijing, China) by microwave heating for 20 min, followed by cooling to room temperature. After washing with PBS, the sections were incubated with 3% hydrogen peroxide for 10 min at room temperature to block endogenous peroxidase activity. The sections were then blocked with blocking solution (Cat# G2052, Servicebio, Wuhan, China) for 30 min and incubated overnight at 4 °C with primary antibodies against CD86 (Cat# DF6332, Affinity, Jiangsu, China) or CD206 (Cat# DF4149, Affinity, Jiangsu, China), both diluted 1:200. After PBS washing, the sections were incubated with ready-to-use HRP-conjugated secondary antibody (Cat# RGAR011, Proteintech, Wuhan, China) for 45 min at room temperature. Signal was developed using DAB substrate (Cat# PR30018, Proteintech, Wuhan, China), followed by hematoxylin counterstaining (Cat# G1120, Solarbio, Beijing, China). Finally, the sections were dehydrated through graded ethanol, cleared in xylene, mounted with neutral resin, and imaged using an upright microscope (BX53F2C, Olympus).

### 2.13 Biochemical analysis of plasma

Plasma was obtained by centrifuging whole blood at 12,000 rpm for 10 min at 4 °C. Alanine aminotransferase (ALT) and aspartate aminotransferase (AST) activities were measured using commercial kits (Jiancheng, Cat# C009-3-1 and C010-3-1, Nanjing, China) according to the manufacturer’s protocols.

### 2.14 Metagenomic sequencing

Fecal DNA was extracted and subjected to metagenomic sequencing on the Illumina NovaSeq 6,000 platform. A total of 18 fecal samples were included in the analysis, comprising 10 samples from the EMs group and 8 samples from the EMs+Flu group. The average sequencing depth was approximately 7 GB per sample in the EMs group and 9 GB per sample in the EMs+Flu group. Taxonomic annotation was performed to generate abundance profiles from domain to species levels. Relative abundance profiles were used for community composition analysis, taxonomic bar plots, *α*-diversity, *β*-diversity, and principal coordinates analysis (PCoA). Differential taxa between groups were screened using the Wilcoxon rank-sum test and LEfSe analysis, which were used as exploratory approaches to identify candidate taxa associated with Flu treatment. To further control for multiple testing, *p* values from differential abundance analyses were adjusted using the Benjamini–Hochberg false discovery rate (FDR) method, and the adjusted results are provided in Table S1. Sequencing and bioinformatics analyses were conducted by APTBIO Co., Ltd. (Shanghai, China).

### 2.15 Fecal DNA extraction and quantitative PCR analysis

Total genomic DNA was extracted from mouse fecal samples using a commercial fecal DNA extraction kit (Tiangen, DP328-02, Beijing, China) according to the manufacturer’s instructions. Total bacterial load was evaluated by qPCR targeting the universal bacterial 16S rRNA gene (primers 338F/518R) (Mohammed et al., 2022). Relative total bacterial abundance was calculated using the 2^-ΔΔCt method (Han et al., 2020; Samarra et al., 2024), with the EMs group serving as the calibrator. The relative abundance of *Akkermansia muciniphila* was determined using species-specific primers and normalized to total bacterial 16S rRNA gene Ct values. Relative changes between groups were calculated using the 2^-ΔΔCt method, with the EMs group serving as the calibrator. All qPCR reactions were performed under the same conditions as described above. Primer sequences are listed in Table 1.

### 2.16 Statistical analysis

Data are presented as mean ± SEM. Statistical analyses were performed using GraphPad Prism. Normality was assessed within each group using the Shapiro–Wilk test. For comparisons between two groups, Welch’s t-test was used when data were normally distributed. If normality assumptions were not satisfied, the Mann–Whitney U test was applied. For comparisons among three or more groups, one-way ANOVA followed by Tukey’s *post hoc* test was used when normality assumptions were reasonably satisfied and variances were homogeneous. Homogeneity of variances was evaluated using the Brown–Forsythe test. If variance heterogeneity was detected, Welch’s ANOVA was applied. When normality assumptions were not satisfied for multi-group comparisons, the Kruskal–Wallis test was used. A *p* value < 0.05 was considered statistically significant. Graphical illustrations were created using BioRender.

## 3 Results

### 3.1 Oral administration of fluvastatin alleviated endometriosis

**Figure 1:**
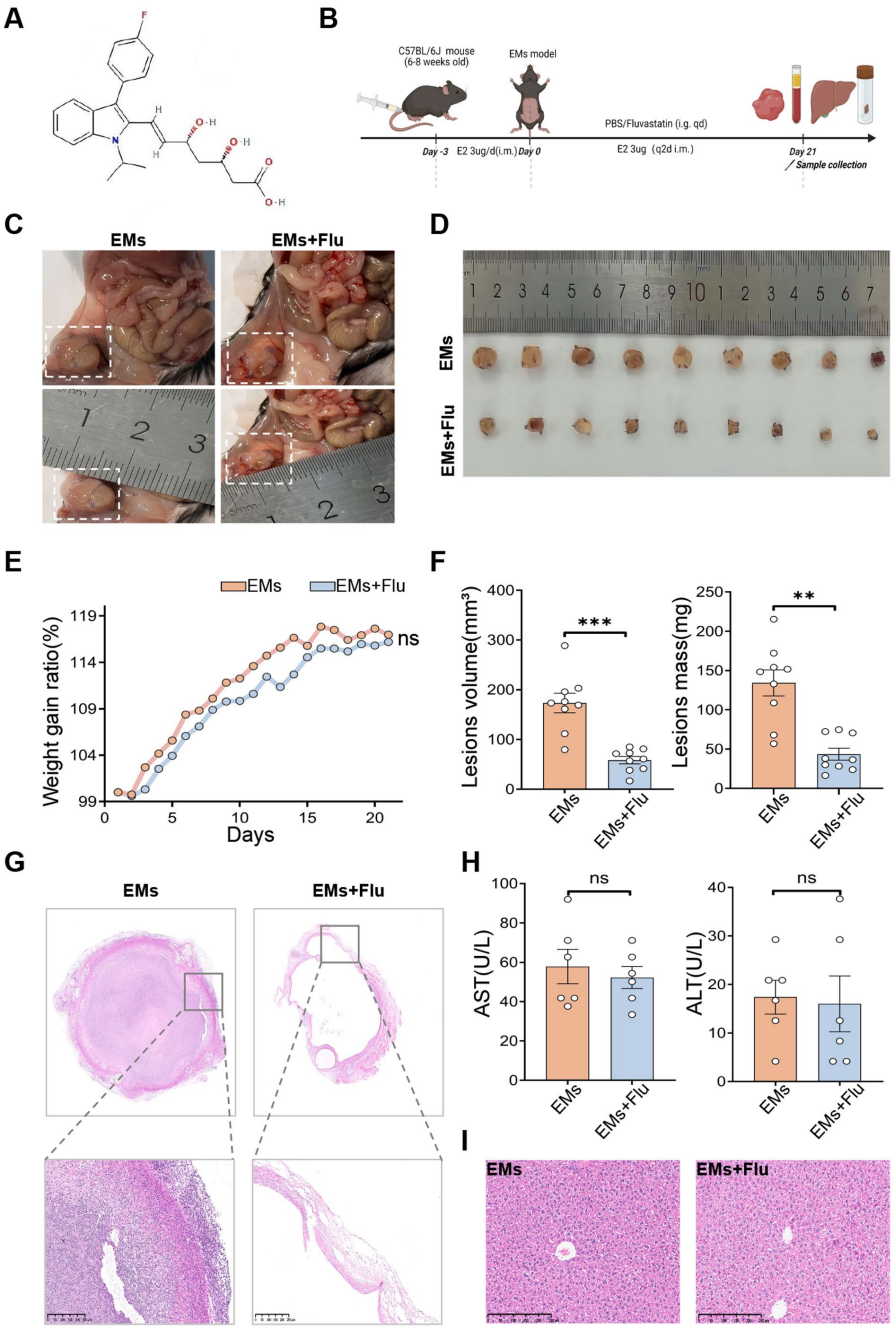
Fluvastatin inhibited endometriosis in mice without obvious toxicity. **(A)** The 2D molecular structure of Flu was sourced from PubChem. **(B)** Experimental scheme. Mice were gavaged daily with PBS or Flu (50 mg/kg body weight) for 21 days following EMs induction. **(C,D)** Representative macroscopic images of ectopic lesions from different treatment groups (*n* = 9). **(E)** Dynamic changes in average body weight across groups (*n* = 6). **(F)** Volume (left) and mass (right) of ectopic lesions in the EMs and EMs+Flu groups (*n* = 9). **(G)** Representative H&E staining of ectopic lesions in the EMs and EMs+Flu groups (*n* = 3). Scale bar: 250 μm. **(H)** The levels of alanine aminotransferase (ALT) and aspartate aminotransferase (AST) in plasma (*n* = 6). **(I)** Representative H&E-stained images of liver tissue from the EMs and EMs+Flu groups (*n* = 3). Scale bar: 250 μm. Data are presented as mean ± SEM. Statistical analysis was performed using Welch’s *t*-test or Mann–Whitney U test (two groups) and one-way ANOVA with Tukey’s *post hoc* test, Welch’s ANOVA, or Kruskal–Wallis test as appropriate (three or more groups). *p < 0.05, **p < 0.01, ***p < 0.001.

To assess the potential of Flu (Figure 1A) in alleviating EMs, we established an EMs mouse model using autologous endometrial transplantation. Mice in the EMs and EMs+Flu groups received daily oral PBS or Flu, respectively (Figure 1B). On day 21, mice were euthanized, and ectopic lesions in the peritoneal cavity were collected for analysis. Relative to the untreated model group (EMs), Flu treatment significantly inhibited the growth of ectopic lesions (Figures 1C,D), as indicated by reductions in both lesion volume and mass (Figure 1F). Hematoxylin and eosin (H&E) staining further showed that Flu treatment reduced endometrial thickness and glandular hyperplasia (Figure 1G). Considering the potential hepatotoxicity of statins, we evaluated the safety of Flu. We monitored body weight throughout the experiment and collected plasma and liver tissue for biochemical and histological analysis. The weight of both groups of mice increased steadily, and no significant difference was observed (Figure 1E). The plasma levels of alanine transaminase (ALT) and aspartate transaminase (AST) did not differ significantly between the EMs+Flu and EMs groups (Figure 1H). In addition, the H&E staining of the liver section showed that the lobular structure in the EMs+Flu group was well preserved, the hepatocyte cords were intact, and there were no obvious signs of fat degeneration or necrosis (Figure 1I). Our findings showed that Flu treatment reduced the growth of ectopic lesions without causing obvious liver toxicity.

### 3.2 Fluvastatin was associated with reduced immune imbalance in endometriosis

**Figure 2:**
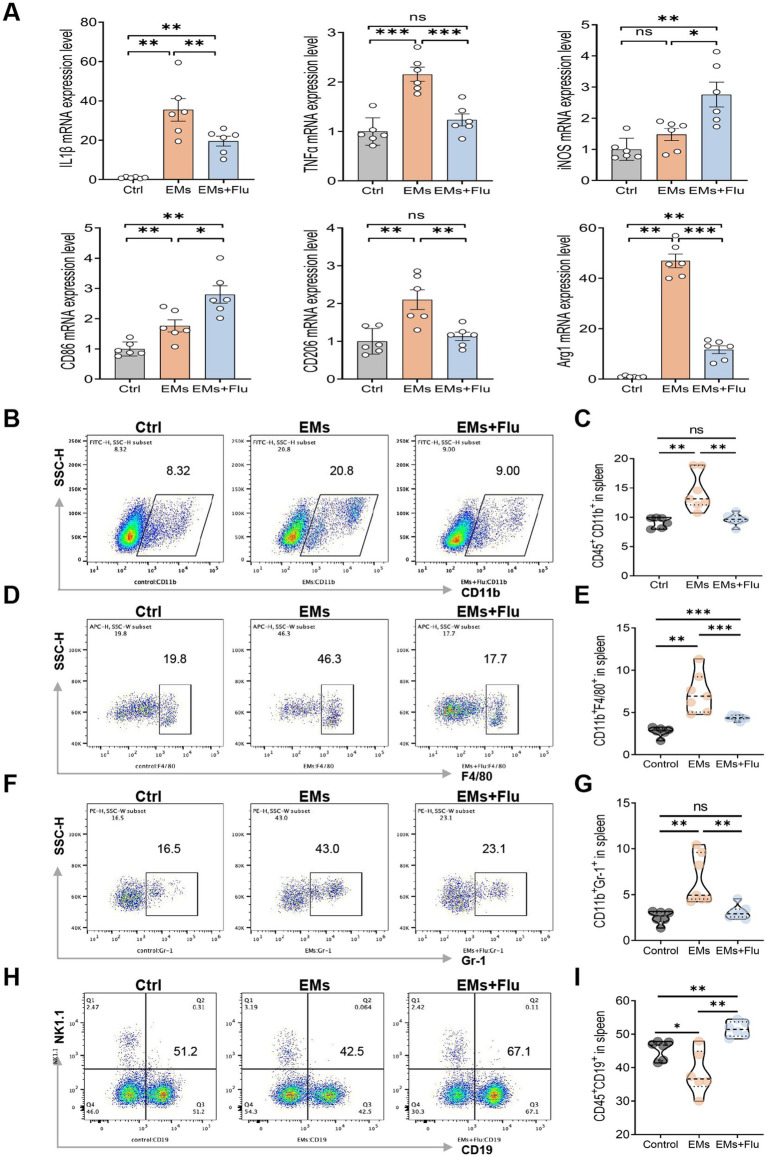
Fluvastatin modulated immune cell populations and macrophage-related markers in endometriosis. **(A)** Relative mRNA expression of cytokines involved in inflammation and markers of macrophage polarization in uterine horns and ectopic lesions: IL-1β, TNF-*α*, iNOS, CD86, CD206, and Arg1 (*n* = 6). **(B–I)** Flow cytometric analysis of splenic immune cells. **(B)** Representative contour plot of CD11b^+^ myeloid cells within CD45^+^cells; **(C)** quantification. **(D)** Representative contour plot of F4/80^+^ macrophages within CD11b^+^cells; **(E)** quantification. **(F)** Representative contour plot of Gr-1^+^ myeloid cells within CD11b^+^cells; **(G)** quantification (*n* = 6, 7, 7). **(H)** Representative contour plot of CD19^+^ B cells within CD45^+^cells; **(I)** quantification (*n* = 6). Data are presented as mean ± SEM. Statistical analysis was performed using Welch’s *t*-test or Mann–Whitney U test (two groups) and one-way ANOVA with Tukey’s post hoc test, Welch’s ANOVA, or Kruskal–Wallis test as appropriate (three or more groups). *p < 0.05, **p < 0.01, ***p < 0.001.

Recent studies have shown that EMs is a chronic systemic disorder rather than a purely localized disease (Hugh S Taylor et al., 2021). Its pathogenesis is complex, and inflammation and immune response are among the most widely accepted hypotheses (Wang et al., 2020). In EMs, disease progression has been associated with dysregulated macrophage polarization, often characterized by enrichment of M2-like signatures within lesions and the peritoneal environment (Gou et al., 2019; Ono et al., 2021; Xu et al., 2025; Dai et al., 2025; Li et al., 2021). After we found that Flu could safely and effectively inhibit the growth of ectopic lesions, we further explored whether the EMs mouse model showed systemic immune imbalance, and whether Flu had anti-inflammatory and immunomodulatory effects. We first analyzed the immune cell populations in the spleen using flow cytometry. Our results demonstrated that, compared to the control mice (Ctrl), the proportion of total myeloid cells (CD45^+^CD11b^+^), granulocytic myeloid cells (CD11b^+^Gr-1^+^), and macrophages (CD11b^+^F4/80^+^) in the spleen of EMs mice was significantly increased. After treatment with Flu, the level of these immune cells were reduced and shifted toward control levels (Figures 2B–G), suggesting that Flu may alleviate immune over-activation. In addition, we observed an increase in the proportion of splenic B lymphocytes (CD45^+^CD19^+^) in Flu-treated mice (Figures 2H,I), suggesting that the immunological changes associated with Flu treatment may involve multiple immune cell populations. The proportions of splenic CD3^+^CD4^+^ T cells, CD3^+^CD8^+^ T cells, and CD45^+^NK1.1^+^ cells were analyzed, and no statistically significant differences were observed between groups (Supplementary Figure S2A–E). To further evaluate its impact on the local immune environment, we examined pro-inflammatory cytokines expression and macrophage polarization markers in uterine horns and ectopic lesions. In comparison to the Ctrl group, the mRNA levels of IL-1β and TNF-*α* in the EMs group were significantly increased, showing that the inflammatory response was enhanced. At the same time, macrophage polarization was skewed toward the M2 phenotype, as indicated by increased expression of CD206 and Arg1 and decreased expression of M1 markers such as iNOS and CD86. In contrast, in the EMs+Flu group, IL-1β and TNF-*α* expression levels were markedly reduced. Flu treatment also upregulated the M1 markers iNOS and CD86 while downregulating the M2 markers CD206 and Arg1 (Figure 2A). Overall, these data suggest that Flu treatment was associated with reduced systemic inflammation and immune abnormalities in EMs model mice, accompanied by changes in splenic immune cell composition, pro-inflammatory cytokine expression, and macrophage-related markers.

### 3.3 Gut microbiota contributes to the protective effects of fluvastatin in endometriosis

**Figure 3:**
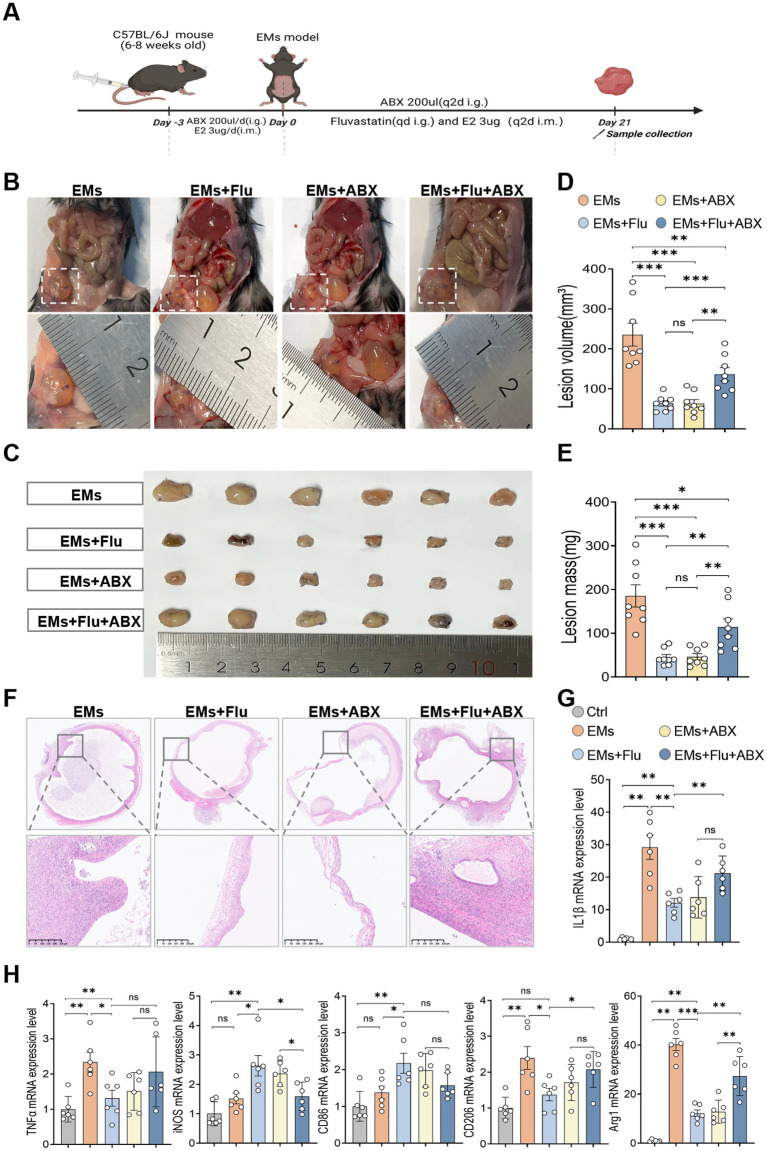
Gut microbiota depletion attenuated the protective changes associated with fluvastatin treatment in endometriosis. **(A)** Schematic diagram of the experimental design. **(B,C)** Representative macroscopic images of ectopic lesions across treatment groups (*n* = 6). **(D,E)** Lesion volume and mass in the EMs, EMs+Flu, EMs+ABX, and EMs+Flu+ABX groups (*n* = 8). **(F)** Representative H&E-stained images of ectopic lesions from each group (*n* = 3). Scale bar: 250 μm. **(G,H)** Relative mRNA expression levels of pro-inflammatory factors and macrophage polarization-associated marker genes in the uterine horns and ectopic lesions: IL-1β, TNF-α, iNOS, CD86, CD206, and Arg1 (*n* = 6). Data are presented as mean ± SEM. Statistical analysis was performed using Welch’s *t*-test or Mann–Whitney U test (two groups) and one-way ANOVA with Tukey’s *post hoc* test, Welch’s ANOVA, or Kruskal–Wallis test as appropriate (three or more groups). *p < 0.05, **p < 0.01, ***p < 0.001.

Previous studies have reported that statins can interact with GM, thereby exerting anti-inflammatory and immunomodulatory effects (Libby, 2020; Vieira-Silva et al., 2020; She et al., 2024). To determine whether GM contributed to the protective effect of Flu in EMs, we used an ABX to deplete endogenous gut microbes in mice. qPCR analysis of the universal bacterial 16S rRNA gene showed an approximately 333-fold reduction in fecal bacterial load in the EMs+ABX group compared with the EMs group, supporting effective microbiota depletion by the ABX (Supplementary Figure S3A). After microbial depletion, EMs were induced surgically. On the day following EMs induction, mice were randomly assigned to four groups: EMs, EMs+Flu, EMs+ABX, and EMs+Flu+ABX. Mice in the EMs+Flu and EMs+Flu+ABX groups received daily oral Flu (50 mg/kg, 200 μL) for 21 days, whereas the other groups received an equivalent volume of PBS by gavage (Figure 3A). In addition, all ABX-treated groups continued to receive antibiotics by oral gavage every other day throughout the experiment. Consistent with our previous findings, the EMs+Flu group showed significantly reduced ectopic lesion size compared with the EMs group (Figures 3B–E). Interestingly, ABX treatment alone (EMs+ABX group) produced a similar reduction in lesion severity (Figures 3B–E). Both the EMs+Flu and EMs+ABX groups displayed reduced mRNA expression of the pro-inflammatory cytokines IL-1β and TNF-*α*, increased expression of M1 macrophage markers (iNOS and CD86), and decreased expression of the M2 markers CD206 and Arg1 compared with the EMs group (Figures 3G,H). To further determine whether these macrophage-related changes were present locally within ectopic lesions and at the protein level, we performed immunofluorescence and immunohistochemical staining for CD86 and CD206 in lesion tissues. Compared with the EMs group, the EMs+Flu and EMs+ABX groups showed increased CD86 expression and decreased CD206 expression (Supplementary Figures S4A,B), consistent with the qPCR results. H&E staining revealed decreased epithelial and stromal components and reduced glandular density in ectopic lesions in both the EMs+Flu and EMs+ABX groups (Figure 3F). However, in the EMs+Flu+ABX group, in which endogenous GM had been depleted, the protective effects associated with Flu treatment were markedly reduced (Figures 3B,C). Lesion volume and mass were significantly higher than those in the EMs+Flu group (Figures 3D,E). Histological analysis showed thicker endometrial tissue and more pronounced inflammatory cell infiltration (Figure 3F). qPCR analysis revealed increased mRNA levels of IL-1β, TNF-*α*, CD206, and Arg1, accompanied by downregulation of iNOS and CD86 (Figures 3G,H). Immunofluorescence and immunohistochemical analyses further showed that, compared with the EMs+Flu group, the EMs+Flu+ABX group exhibited increased CD206 expression and decreased CD86 expression in ectopic lesions (Supplementary Figures S4A,B). Together, these findings suggest that an intact GM may contribute to, but may not fully account for, the protective effects associated with Flu treatment in the EMs model. To further evaluate whether the ABX alters baseline immune status in the absence of EMs induction, we performed an additional short-term experiment in normal mice (Ctrl vs. Ctrl+ABX) and analyzed splenic immune cells by flow cytometry. The results showed that, under non-EMs conditions, ABX treatment did not result in statistically significant changes in the proportions of major splenic immune cell populations (Supplementary Figures S3C–L).

### 3.4 Transplanting the fecal microbiota from fluvastatin-treated mice partially reproduced protective changes in endometriosis

**Figure 4:**
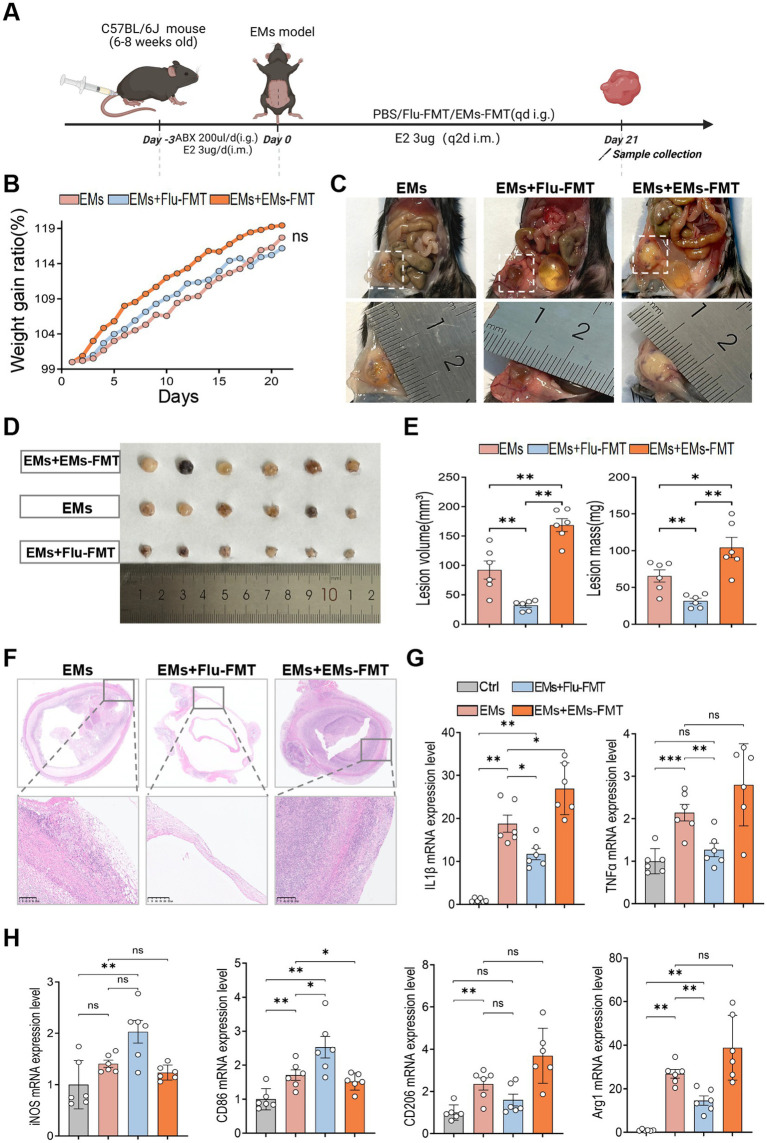
Fecal microbiota from fluvastatin-treated mice reduced lesion burden and inflammatory changes in endometriosis. **(A)** Diagrammatic representation of the experimental design. **(B)** Dynamic changes in average body weight among treatment groups (*n* = 6). **(C,D)** Representative gross images of ectopic lesions in mice from different treatment groups (*n* = 6). **(E)** Volume (left) and mass (right) of ectopic lesions in the EMs, EMs+Flu-FMT, and EMs+EMs-FMT groups (*n* = 6). **(F)** Representative H&E-stained images of ectopic lesions from each group (*n* = 3). Scale bar: 250 μm. **(G,H)** Relative mRNA expression levels of pro-inflammatory factors and macrophage polarization-related marker genes in the uterine horns and ectopic lesions: IL-1β, TNF-α, iNOS, CD86, CD206, and Arg1 (*n* = 6). Data are presented as mean ± SEM. Statistical analysis was performed using Welch’s t-test or Mann–Whitney U test (two groups) and one-way ANOVA with Tukey’s post hoc test, Welch’s ANOVA, or Kruskal–Wallis test as appropriate (three or more groups). *p < 0.05, **p < 0.01, ***p < 0.001.

To further evaluate the involvement of GM in Flu-mediated protection against EMs, we performed FMT using donor feces from Flu-treated mice. Recipient EMs mice were pretreated with antibiotics to deplete endogenous GM and then subjected to FMT. Mice were randomly allocated to three groups: EMs (receiving PBS), EMs+Flu-FMT (receiving fecal microbiota from EMs+Flu donors), and EMs+EMs-FMT (receiving microbiota from EMs donors) (Figure 4A). Disease severity was significantly reduced in the EMs+Flu-FMT group compared with the EMs group (Figures 4C,D). Lesion volume and mass were significantly reduced following FMT from Flu-treated donors (Figure 4E). H&E staining showed reduced endometrial thickening and decreased inflammatory cell infiltration in the EMs+Flu-FMT group (Figure 4F). In line with these findings, qPCR analysis revealed reduced expression of pro-inflammatory cytokines IL-1β and TNF-α, as well as M2 macrophage markers CD206 and Arg1, in the EMs +Flu-FMT group (Figures 4G,H). Conversely, M1 macrophage markers iNOS and CD86 were significantly upregulated (Figures 4G,H). To further determine whether these changes were also present locally within ectopic lesions and at the protein level, we performed immunofluorescence and immunohistochemical staining for CD86 and CD206 in lesion tissues. Compared with both the EMs and EMs+EMs-FMT groups, the EMs+Flu-FMT group showed increased CD86 expression and decreased CD206 expression (Supplementary Figures S4C,D), consistent with the qPCR results. These findings suggest that fecal microbiota from Flu-treated mice was associated with local macrophage-related changes within ectopic lesions. In contrast, mice receiving fecal bacteria from EMs mice (EMs+EMs-FMT) did not exhibit protective effects but instead showed increased lesion severity (Figures 4C–H). Throughout the experiment, no significant differences in body weight were detected across the groups (Figure 4B). Overall, these results suggest that FMT using microbiota from Flu-treated donors partially reproduced the protective changes associated with Flu treatment in EMs. This further supported the idea that microbiota altered by Flu may contribute to, but may not fully explain, its protective effects.

### 3.5 Fluvastatin altered gut microbiota composition and was associated with increased abundance of *Akkermansia muciniphila*

**Figure 5:**
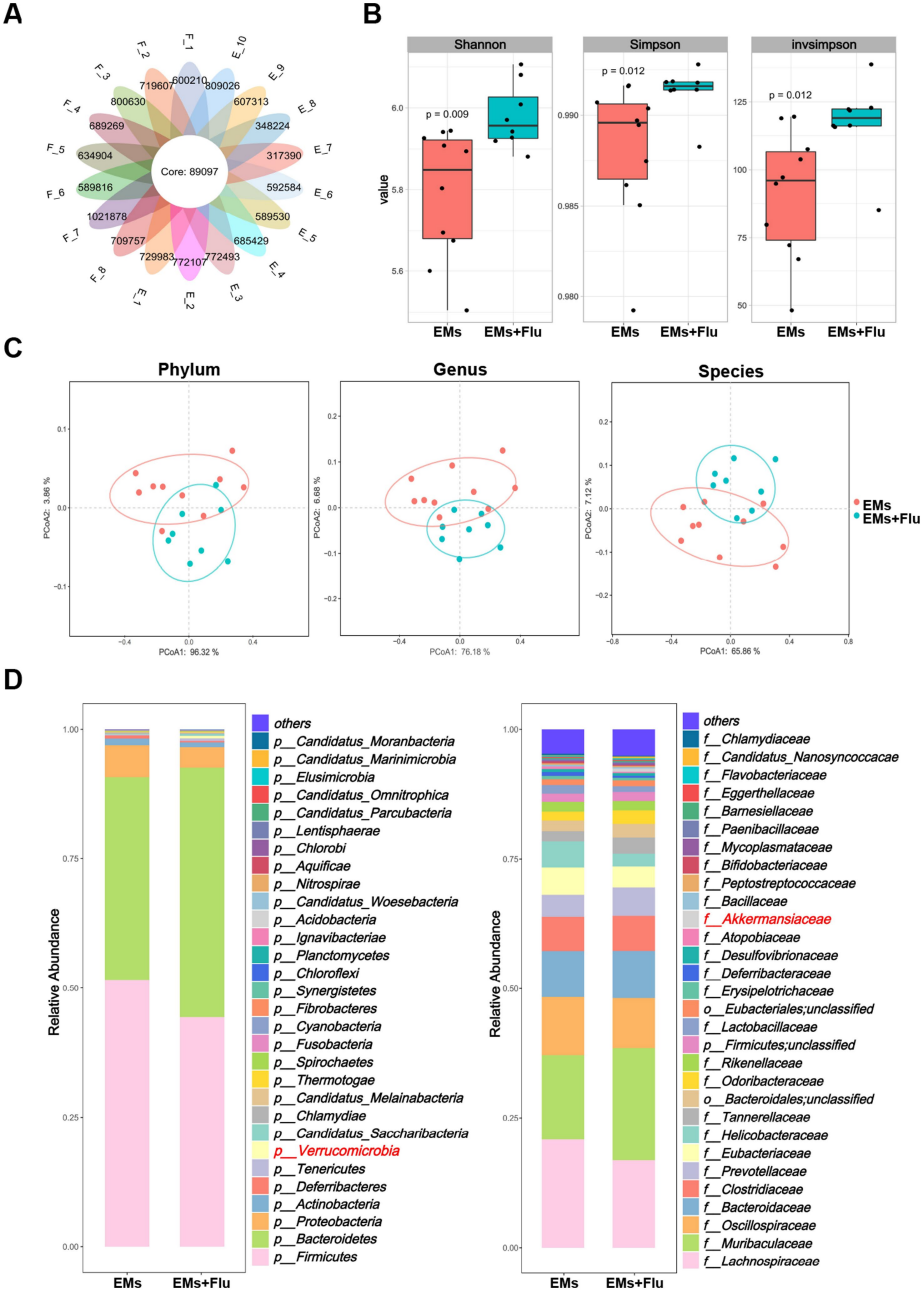
Fluvastatin altered gut microbiota composition in endometriosis mice. **(A)** Venn diagram shows the number of shared and unique genes between the EMs and EMs+Flu (*n* = 10, 8). **(B)** Alpha diversity indices (Shannon, Simpson, and inverse Simpson) comparing the EMs and EMs+Flu groups. **(C)** PCoA based on Bray–Curtis distances, illustrating beta diversity at the phylum, genus, and species levels. **(D)** Bar charts displaying relative abundance of GM at the phylum and family levels. Data are presented as mean ± SEM. Statistical analysis was performed as described in the Methods section. *p < 0.05.

**Figure 6:**
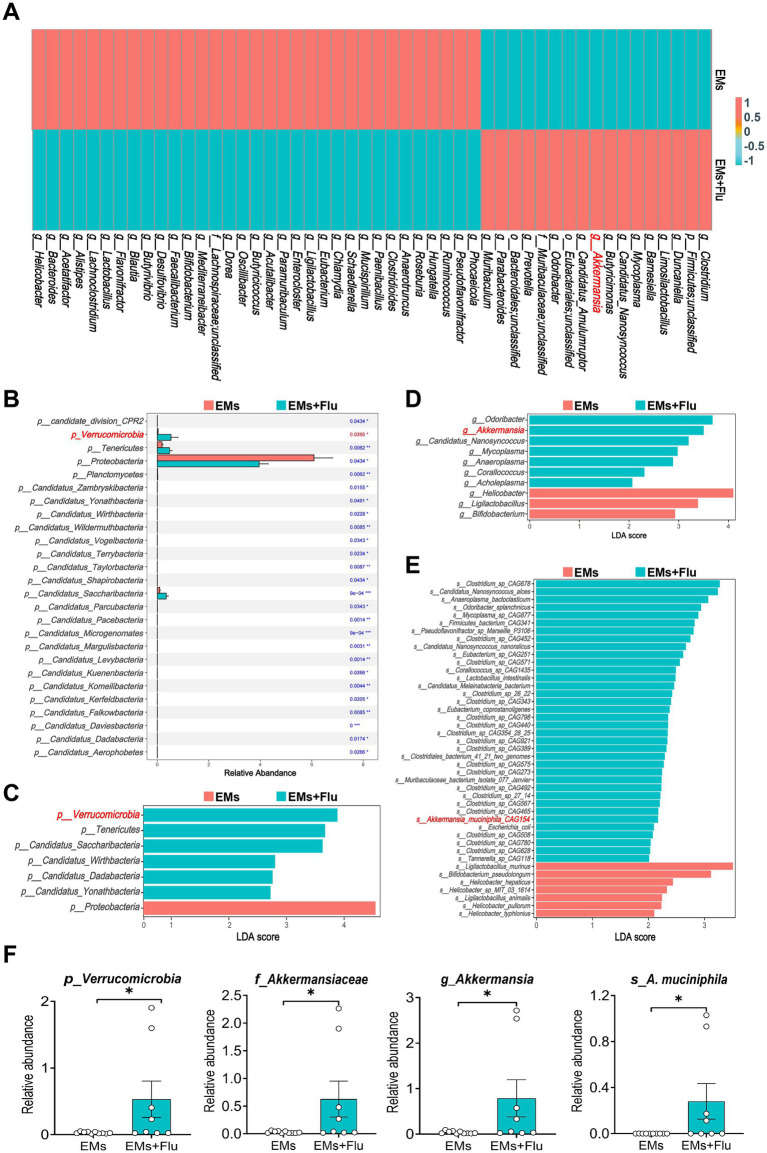
Fluvastatin increased the abundance of *Akkermansia muciniphila* in endometriosis mice. **(A)** Species abundance cluster heatmap (displaying the top 50 species by genus level). Color intensity reflects abundance, highlighting community structure differences between groups. **(B)** Differential abundance analysis at the phylum level between groups. **(C–E)** Linear discriminant analysis with effect size (LEfSe) identifying significantly different microbial taxa between EMs and EMs+Flu groups at the phylum, genus, and species levels. **(F)** Quantification of the relative abundance of *Verrucomicrobia* (phylum), *Akkermansiaceae* (family), *Akkermansia* (genus), and *A. muciniphila* (species). Data are presented as mean ± SEM. Statistical analysis was performed as described in the Methods section. *p < 0.05.

To investigate how Flu influences the GM, we performed metagenomic sequencing on fecal samples from EMs mice with or without Flu treatment. A Venn diagram was generated to compare shared and unique microbial genes between the groups (Figure 5A). Alpha diversity results indicated that Flu treatment altered GM richness and diversity relative to the EMs group (Figure 5B). Principal coordinates analysis (PCoA) based on Bray–Curtis distance further showed a clear separation in community structure between the two groups (Figure 5C), suggesting notable microbiota shifts. Taxonomic bar plots illustrated an increased proportion of *Verrucomicrobia* in the EMs+Flu group, accompanied by reduced *Firmicutes* and *Proteobacteria* (Figure 5D), with similar trends observed at the family level. A heatmap of the top 50 genera showed higher abundance of *A. muciniphila,* and lower levels of opportunistic genera such as *Helicobacter* and *Bacteroides* (Figure 6A). Consistent with these findings, differential abundance analysis at the phylum level showed that *Verrucomicrobia* was enriched in the EMs+Flu group at the nominal significance level (*p* < 0.05, Figure 6B). To identify taxa most associated with Flu treatment, we conducted LEfSe analysis (Figures 6C–E). The taxa enriched in the EMs+Flu group were primarily derived from *Verrucomicrobia*, including *A. muciniphila,* with increases observed from the phylum down to the species level. Conversely, microbes such as *Helicobacter* (*Proteobacteria*) were reduced. *A. muciniphila*, a dominant species within *Verrucomicrobia*, showed an increased relative abundance in the EMs+Flu group (Figure 6F). Species-specific qPCR analysis further validated this finding, demonstrating that the abundance of *A. muciniphila* was significantly higher in the EMs+Flu group than in the EMs group. Consistent results were obtained in an independent cohort of mice, supporting the reproducibility of this observation (Supplementary Figure S3B). It should be noted that, after strict multiple-testing correction, only a limited number of taxa remained statistically significant in the differential abundance analysis (Supplementary Table S1). Therefore, the metagenomic results are better interpreted as an exploratory approach for identifying candidate taxa rather than as definitive mechanistic evidence. Based on the sequencing results and species-specific qPCR validation, we considered *A. muciniphila* as a candidate functional bacterium worthy of further investigation, rather than the sole or primary mediator of Flu-associated effects. Previous studies have reported its health-promoting effects and potential as a next-generation probiotic (Xie et al., 2023). Taken together, these findings suggest that Flu altered the GM of EMs mice and was associated with increased abundance of candidate functional bacteria, including *A. muciniphila*. These microbiota changes may contribute to the protective effects associated with Flu treatment.

### 3.6 *Akkermansia muciniphila* supplementation was associated with protective changes in endometriosis

**Figure 7:**
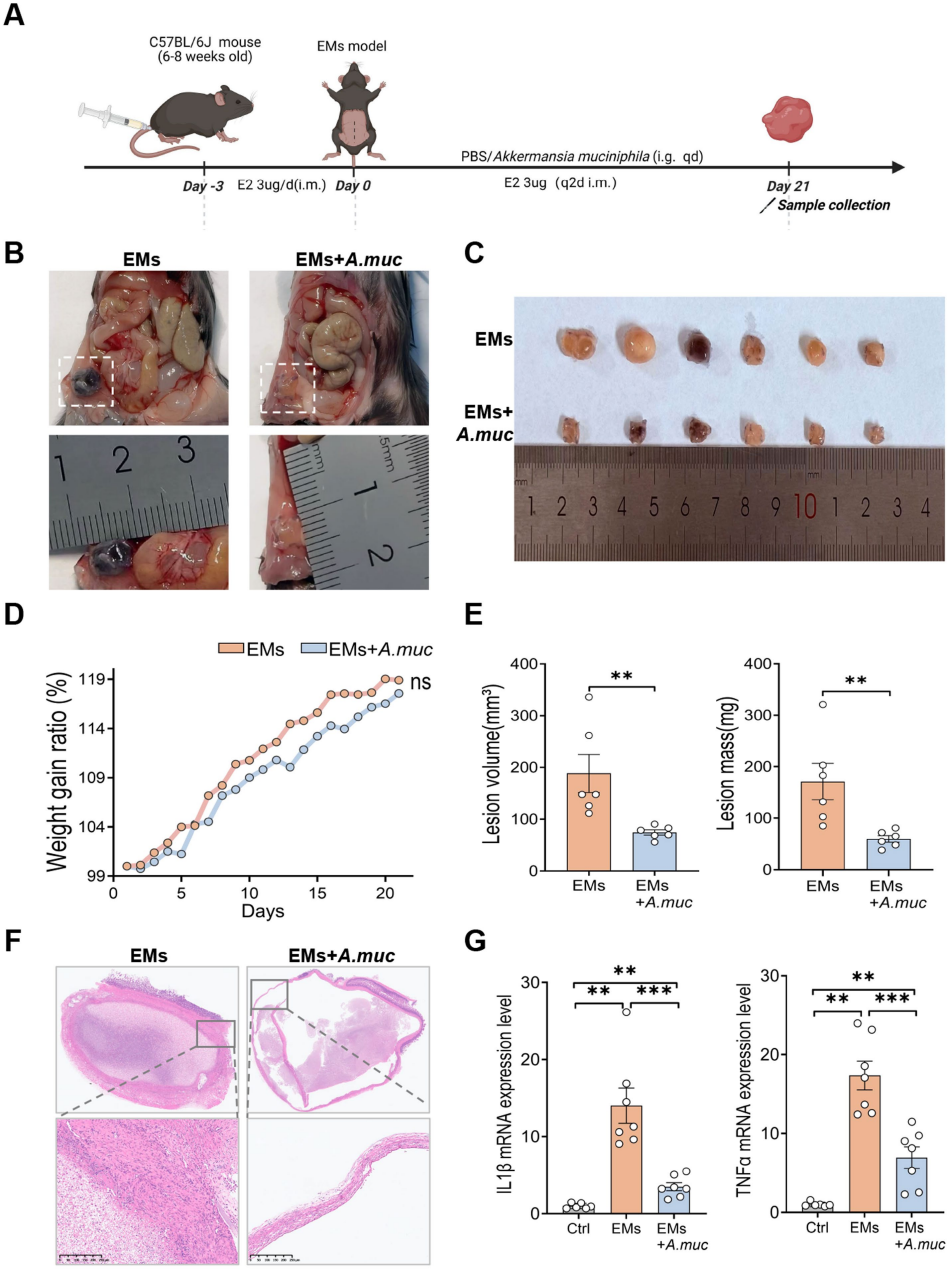
*Akkermansia muciniphila* supplementation reduced lesion burden and inflammatory changes in endometriosis mice. **(A)** Experimental scheme: Mice were administered daily oral gavage of sterile PBS (vehicle control) or *A. muciniphila* (1.5 × 10^9^ CFU/mouse) for 21 days starting the day after EMs induction. **(B,C)** Representative *in vivo* and *ex vivo* images of ectopic lesions in EMs and EMs+*A.muc* groups (*n* = 6). **(D)** Dynamic variations in average body weight among the groups (*n* = 6). **(E)** Quantification of lesion volume (left) and mass (right) in the EMs and EMs+*A.muc* groups (*n* = 6). **(F)** Representative H&E-stained images of ectopic lesions (*n* = 3). Scale bar: 250 μm. **(G)** qPCR analysis of inflammatory mediator’s relative mRNA expression levels in uterine horns and ectopic lesions (*n* = 6, 7, 7). Data are presented as mean ± SEM. Statistical analysis was performed using Welch’s *t*-test or Mann–Whitney *U* test (two groups) and one-way ANOVA with Tukey’s post hoc test, Welch’s ANOVA, or Kruskal–Wallis test as appropriate (three or more groups). *p < 0.05, **p < 0.01, ***p < 0.001.

**Figure 8:**
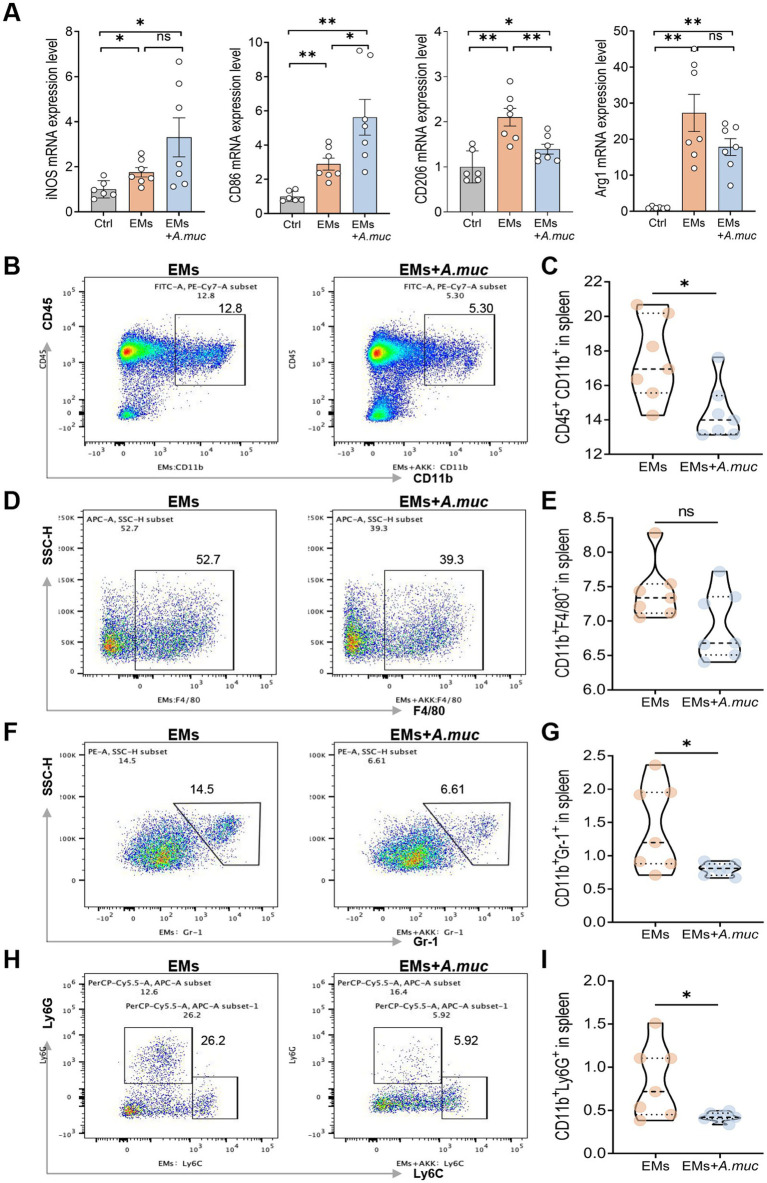
*Akkermansia muciniphila* attenuated endometriosis by modulating immune responses. **(A)** Relative mRNA expression levels of macrophage polarization markers in the uterine horns and ectopic lesions: iNOS, CD86, CD206, and Arg1 (*n* = 6, 7, 7). **(B–I)** Flow cytometric analysis of splenic immune cell populations. **(B,C)** Representative contour plot and quantification of CD11b^+^ myeloid cells within CD45^+^cells. **(D,E)** Representative contour plot and quantification of F4/80^+^ macrophages within CD11b^+^cells. **(F,G)** Representative plot and quantification of Gr-1^+^ myeloid cells within CD11b^+^cells. **(H,I)** Representative plot and quantification of Ly6G^+^ neutrophils within CD11b^+^cells (*n* = 7). Data are presented as mean ± SEM. Statistical analysis was performed using Welch’s *t*-test or Mann–Whitney U test (two groups) and one-way ANOVA with Tukey’s post hoc test, Welch’s ANOVA, or Kruskal–Wallis test as appropriate (three or more groups). *p < 0.05, **p < 0.01, ***p < 0.001.

Based on the metagenomic and qPCR findings, we selected *A. muciniphila* for further investigation as a candidate functional bacterium associated with Flu treatment. EMs model mice were therefore orally gavaged with *A. muciniphila* at a dose of 1.5 × 10^9^ CFU for 21 days (Figure 7A), and body weight of the mice was monitored throughout the experimental period, showing no significant difference between the EMs and EMs+A.muc groups (Figure 7D). This was followed by lesion assessment (Figures 7B,C). The average lesion volume in the EMs group was 188.51 mm^3^, compared with 74.50 mm^3^ in the EMs+*A.muc* group. Similarly, average lesion mass decreased from 171.17 mg in the EMs group to 59.83 mg in the treatment group (*p* < 0.01, Figure 7E). H&E staining revealed marked glandular hyperplasia in the EMs group, whereas *A. muciniphila*-treated mice exhibited thinner lesion capsules and reduced hyperplasia (Figure 7F). qPCR results showed that *A. muciniphila* treatment significantly downregulated pro-inflammatory cytokines, along with M2 macrophage markers CD206 and Arg1 (Figure 7G). Conversely, the levels of the M1 macrophage markers iNOS and CD86 were markedly elevated (Figure 8A). Immunofluorescence and immunohistochemical analyses further showed that the EMs+*A.muc* group had increased CD86 expression and decreased CD206 expression in ectopic lesions compared with the EMs group (Supplementary Figures S4C,D). Flow cytometry of splenic immune cells further confirmed a reduction in the proportions of activated myeloid cells (CD45^+^CD11b^+^), granulocytes (CD11b^+^Gr-1^+^), neutrophils (CD11b^+^Ly6G^+^), and macrophages (CD11b^+^F4/80^+^) in the EMs+*A.muc* group compared to the EMs group (Figures 8B–I). In addition, in the *A. muciniphila* supplementation experiment, no significant changes were observed in splenic CD45^+^CD19^+^ B lymphocytes or NK1.1^+^ cells (Supplementary Figures S2F–H). Taken together, these results suggest that *A. muciniphila* supplementation was associated with reduced inflammation and macrophage-related changes in the EMs model, supporting its potential role as a candidate functional bacterium associated with the protective effects of Flu treatment. The gating strategies used in this study are detailed in the supplementary information (Supplementary Figure S1).

## 4 Discussion

Microbiota dysbiosis has increasingly been recognized as a research focus in gynecology, given its reported associations with a range of reproductive system disorders (Aquino et al., 2024). Substantial evidence suggests that GM dysbiosis may contribute to the onset and progression of EMs, primarily by inducing chronic inflammation and immune dysregulation (Ansaldo et al., 2021; Yang and Cong, 2021). Although previous studies have suggested GM involvement in EMs, its specific functions remain poorly understood. This study combines antibiotic-mediated microbiota depletion with FMT to provide experimental evidence supporting a contributory role of GM in EMs progression. In our EMs mouse model, antibiotic treatment reduced lesion growth, while microbiota transplantation from EMs mice exacerbated disease in microbiota-depleted recipients. Conversely, transplantation of microbiota from Flu-treated mice produced a protective effect like Flu therapy. These findings suggest that GM may influence EMs progression, providing direction for future microbiota-based therapeutic strategies.

After identifying an association between GM and EMs, we further explored interventions that may influence EMs through changes in GM. Although statins are traditionally used to lower lipids, more studies believe that they can also affect the composition and activity of intestinal flora (Libby, 2020; Vieira-Silva et al., 2020; She et al., 2024). Some studies have indicated that statins may help relieve chronic inflammatory diseases (including EMs) (Qin et al., 2024), but research on the relationship between statins, GM, and EMs is still very limited. Based on this knowledge gap, we focused on Flu, a statin with anti-inflammatory, anti-tumor and immunomodulatory properties (Muffova et al., 2025; Chen et al., 2025; Paragh et al., 2005). Flu also has pharmacokinetic characteristics that are different from other statins and may more easily function locally in the intestine (Yu et al., 2021). Through ABX, FMT, and metagenomic analysis, we observed that the protective effects associated with Flu treatment were accompanied by alterations in GM composition. However, because antibiotic treatment alone reduced lesion burden to a degree comparable to Flu treatment, the current experiments cannot fully distinguish microbiota-dependent effects from the direct systemic or immunomodulatory effects of antibiotics. Therefore, the available data are better interpreted as indicating that GM contributes to, rather than solely mediates, the protective effects associated with Flu treatment.

It is worth noting that *A. muciniphila* remained consistently increased after Flu treatment, suggesting that it may be a candidate functional bacterium associated with the protective effects of Flu treatment. *A. muciniphila* has been proposed as a potential “next-generation probiotic” because it may contribute to maintenance of the mucosal barrier, immune regulation, and inflammatory responses (Cani et al., 2022; Li et al., 2016; Zeng et al., 2023). Previous studies have shown that *A. muciniphila* can induce adaptive immune responses in a physiological state (Ansaldo et al., 2019) and produce biologically active molecules that can regulate host immunity (Yoon et al., 2021). In this study, Flu treatment was associated with increased abundance of *A. muciniphila*, and this change was positively correlated with reduced lesion burden. Direct supplementation with *A. muciniphila* was associated with reduced IL-1β and TNF-*α* expression and with shifts in macrophage-related markers toward a less M2-biased profile. Although the traditional view is that M1 macrophages are inclined toward inflammation and M2 toward immune regulation, in the abdominal microenvironment unique to EMs, M2-biased phenotypes are frequently associated with lesion persistence, whereas rebalancing macrophage polarization may contribute to limiting lesion progression.(Gou et al., 2019; Ono et al., 2021; Li et al., 2021; Park et al., 2025; Dai et al., 2025). In addition, supplementation with *A. muciniphila* was associated with partial normalization of splenic immune cell composition, suggesting a potential role in systemic immune regulation. In summary, these observations suggest that increased abundance of *A. muciniphila* may contribute to the protective effects associated with Flu treatment.

Recent studies emphasize that EMs is not a simple local pelvic disease, but a systemic condition accompanied by systemic inflammation and immune abnormalities (Taylor et al., 2021, Xu et al., 2025). EMs patients often show elevated circulating pro-inflammatory factors (such as IL-6, IL-8, TNF-α) and impaired immune function (Krygere et al., 2024; Wójtowicz et al., 2025). As an important peripheral immune organ, spleen is crucial in maintaining immune homeostasis (Mebius and Kraal, 2005; Bronte and Pittet, 2013). In this study, EMs mice showed increased proportions of splenic myeloid cells and granulocytes in the spleen, and Flu treatment was associated with partial reversal of these changes, suggesting that Flu may affect both local and systemic immune regulation. To better correlate local and systemic immune changes, we tried to analyze the immune cells in the abdominal irrigation solution, but we failed to obtain reliable data due to the insufficient number and activity of cells (Supplementary Figures S2I–K). Although lesion-level immunofluorescence and immunohistochemical analyses supported local changes in macrophage-related markers, future studies should directly characterize infiltrating immune cell subsets within ectopic lesions, for example by flow cytometry or single-cell approaches.

From a therapeutic perspective, microbiota-targeted strategies encompass ecological depletion (e.g., antibiotics that may reduce lesion burden but disrupt microbial homeostasis (Chadchan et al., 2019)), metabolite supplementation (e.g., butyrate), and dietary modulation (Parpex et al., 2024). In this context, Flu represents a strategy that integrates host-directed effects with microbiota-modulating potential. The strengths of the present study include the integration of gut microbiota profiling with systemic immune assessment; evidence supporting a contributory role of the microbiota in disease progression based on antibiotic depletion and fecal microbiota transplantation; supportive evidence from single-strain supplementation with *A. muciniphila*; and the preliminary observation of an association between microbiota modulation and macrophage-related changes within lesions.

This study still has several limitations. First, all experiments were performed in mouse models. Although these models recapitulate several pathological features of human endometriosis, species differences may limit their clinical relevance. Therefore, further validation using human samples, public datasets, and prospective studies will be required. Second, only a single dose of Flu was tested. This dose was selected based on the tumor-like characteristics of EMs (Pearce et al., 2012; Wang et al., 2025a; Varma et al., 2004; Dai et al., 2025) and the established use of Flu in cancer research (Yu et al., 2021; Horiguchi et al., 2004; Kusama et al., 2002; Yang et al., 2017). The intervention period was limited to 21 days. We did not perform systematic dose–response, pharmacokinetic, or time-course analyses. Future studies should therefore include different dose levels, exposure–response analyses, and longer observation periods to determine the minimal effective dose, optimal therapeutic window, and durability of the treatment effect. Third, although the antibiotic depletion, FMT, and *A. muciniphila* supplementation experiments all suggest that the gut microbiota contributes to the protective effects associated with Flu treatment, these findings are not sufficient to establish a strict mechanistic causal relationship. In particular, antibiotic treatment alone reduced lesion burden, and therefore the current experiments cannot fully distinguish microbiota-dependent effects from the direct immunological or metabolic effects of antibiotics themselves. In addition, although *A. muciniphila* showed changes consistent with the Flu-associated phenotype in the metagenomic analysis, species-specific qPCR, and supplementation experiments, it should be interpreted as a candidate functional bacterium rather than as a primary or decisive mediator. The present study did not include heat-killed or bacterial component controls, nor did it systematically compare *A. muciniphila* with other candidate taxa enriched by Flu treatment. Therefore, it remains unclear whether *A. muciniphila* has a specific or exclusive role. Moreover, although *A. muciniphila* supplementation partially improved lesion burden, inflammatory markers, and immune-related parameters, it did not fully reproduce all of the phenotypic and immunological effects observed following Flu treatment. From the perspective of microbiome analysis, we have now provided Benjamini–Hochberg multiple-testing correction for the phylum-level Wilcoxon analysis shown in the main text. However, given the current sample size, most taxa no longer remained significant after strict correction. Therefore, the metagenomic findings should be interpreted as exploratory observations and are not sufficient on their own to support strict taxon-specific or mechanistic conclusions. In addition, this study did not include systematic pathway analysis, metabolomics, or measurements of microbial metabolites. As a result, the functional relationship between microbiota changes and host immune regulation remains unclear. At the immunological level, although we added lesion-level immunofluorescence and immunohistochemical validation of CD86 and CD206, we did not further characterize local immune cell populations within the lesions by flow cytometry, single-cell sequencing, or functional assays. In addition, key T-cell subsets such as Treg and Th17 cells were not further examined. Therefore, the conclusions regarding macrophage polarization and local immune regulation should still be interpreted with caution. Finally, this study mainly focused on the overall effects of Flu in endometriosis. Future studies integrating multi-omics approaches, gene editing, and more detailed immunological analyses will be needed to further clarify the interactions among microbial metabolites, host immunity, and Flu treatment. Given the differences in physicochemical and pharmacokinetic properties among different statins, comparative studies will also be needed to identify agents with the greatest translational potential.

In summary, our findings suggest that Flu treatment was associated with reduced inflammation and immune dysregulation in EMs, accompanied by changes in GM composition, including increased abundance of *A. muciniphila*. This discovery offered a new insight into the potential repurposing of statins in gynecological disorders and offered additional experimental evidence linking statins, GM, and EMs. Considering the limited therapeutic options currently available for EMs, further research into microbiota-informed therapeutic strategies is warranted.

## 5 Conclusion

**Figure 9:**
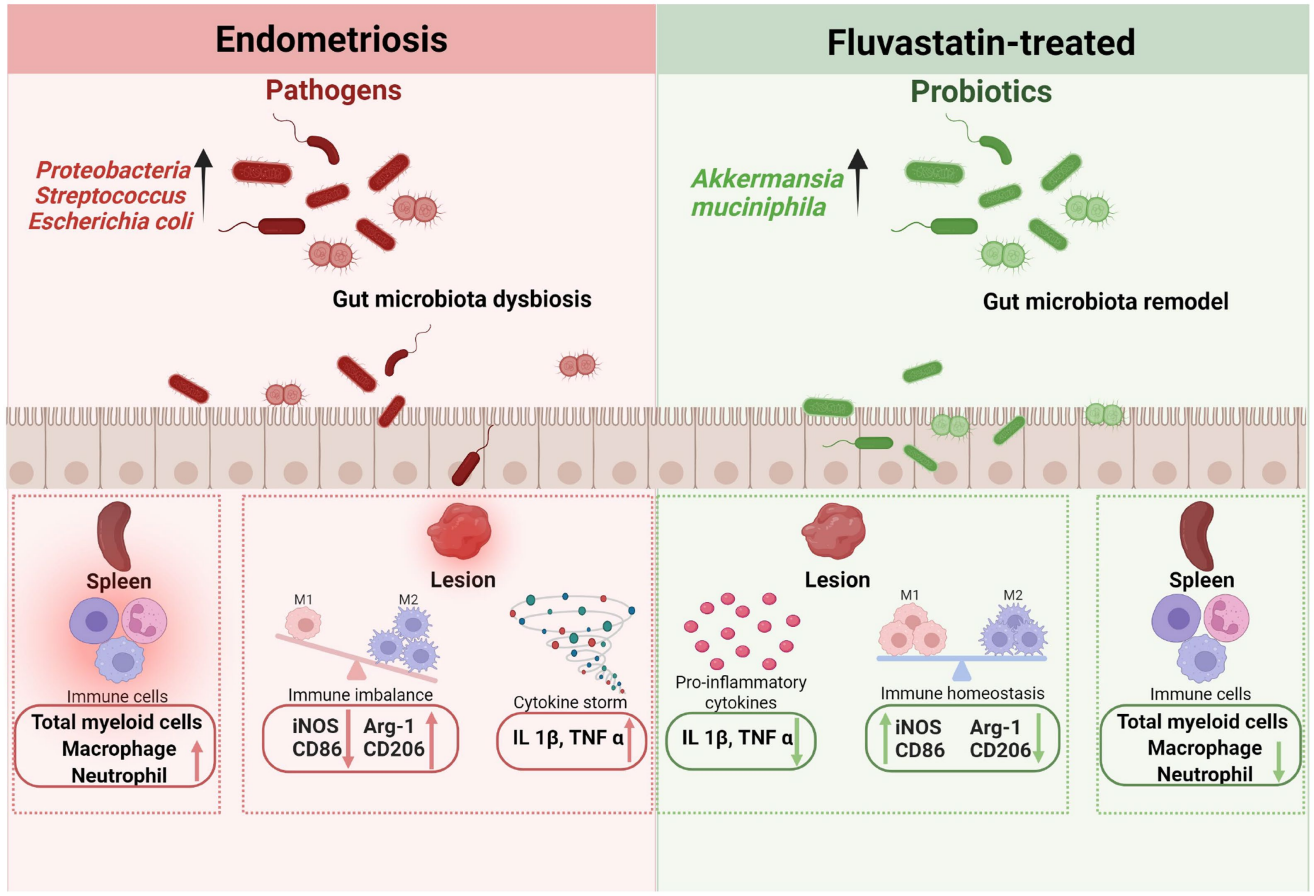
Proposed model of how fluvastatin-associated microbiota changes may contribute to immune regulation in endometriosis. Fluvastatin was associated with changes in gut microbiota composition, including increased abundance of *A. muciniphila*. These changes may contribute to reduced inflammation, altered macrophage-related markers, and improved immune balance in EMs.

In conclusion, our study suggests that Flu treatment was associated with reduced lesion burden, inflammation, and immune dysregulation in EMs, accompanied by changes in gut microbiota composition, including increased abundance of *A. muciniphila* (Figure 9). These findings provide further support for microbiota-informed therapeutic strategies in gynecological disorders and suggest that modulation of the gut microbiota may represent a potential approach for EMs. Additional mechanistic and clinical studies are still needed to further validate these findings and to assess the efficacy and safety of this strategy in patients.

## Data Availability

The metagenomic sequencing data generated in this study have been deposited in NCBI SRA under BioProject accession number PRJNA1457758 (https://www.ncbi.nlm.nih.gov/). Other data supporting the findings of this study are available from the corresponding author upon reasonable request.

## Supplementary material

SUPPLEMENTARY FIGURE S1 Gating strategies used for flow cytometry analysis.

SUPPLEMENTARY FIGURE S2 Flow cytometric analysis of splenic and peritoneal immune cell populations. **(A–E)** Flow cytometric analysis of splenic immune cell populations in Ctrl, EMs, and EMs+Flu groups (*n* = 6, 7, 7). **(A)** Representative flow cytometry plots of T cell subsets. **(B,C)** Quantification of T cell subset proportions. **(D)** Representative flow cytometry plot of NK1.1⁺ cells within CD45⁺ cells. **(E)** Quantification of NK1.1⁺ cell proportions. **(F–H)** Flow cytometric analysis of splenic immune cell populations in EMs and EMs+A. muc groups (*n* = 7). **(F)** Representative flow cytometry plots of CD19⁺ B cells and NK1.1⁺ cells within CD45⁺ cells. **(G,H)** Quantification of the corresponding cell proportions. **(I–K)** Flow cytometric analysis of macrophage markers in peritoneal lavage fluid from Ctrl, EMs, and EMs+Flu groups (*n* = 6, 7, 7). **(I)** Representative flow cytometry plot of CD206⁺ cells. **(J)** Quantification of CD86⁺ cell proportions. **(K)** Quantification of CD206⁺ cell proportions. Data are presented as mean ± SEM. Statistical analysis was performed using Welch’s *t*-test or Mann–Whitney U test (two groups) and one-way ANOVA with Tukey’s *post hoc* test, Welch’s ANOVA, or Kruskal–Wallis test as appropriate (three or more groups). *P < 0.05, ***P* < 0.01, ****P* < 0.001.

SUPPLEMENTARY FIGURE S3 Validation of microbiota depletion, *Akkermansia muciniphila abundance*, and baseline immune status. **(A)** Quantification of total bacterial load (16S rRNA gene) in fecal DNA from EMs and EMs+ABX groups measured by qPCR (*n* = 6). **(B)** Relative abundance of Akkermansia muciniphila in fecal samples from EMs and EMs+Flu groups measured by qPCR at the endpoint of the Flu experiment and in an independent replicate cohort (*n* = 6). **(C–L)** Flow cytometric analysis of splenic immune cell populations in Ctrl and Ctrl+ABX groups (*n* = 6). **(C)** Representative flow cytometry plot of F4/80⁺ macrophages within CD11b⁺ cells. (D) Quantification of F4/80⁺ macrophage proportions. **(E)** Representative flow cytometry plot of Gr-1⁺ granulocytic myeloid cells within CD11b⁺ cells. **(F)** Quantification of Gr-1⁺ cell proportions. **(G)** Representative flow cytometry plots of T cell subsets. **(H,I)** Quantification of T cell subset proportions. **(J)** Representative flow cytometry plots of CD19⁺ B cells and NK1.1⁺ cells within CD3⁻ cells. **(K,L)** Quantification of the corresponding cell proportions. Data are presented as mean ± SEM. Statistical analysis was performed using Welch’s t-test or Mann–Whitney U test (two groups) and one-way ANOVA with Tukey’s *post hoc* test, Welch’s ANOVA, or Kruskal–Wallis test as appropriate (three or more groups). **P* < 0.05, ***P* < 0.01, ****P* < 0.001.

SUPPLEMENTARY FIGURE S4 Changes in CD86 and CD206 expression in ectopic lesions after ABX, FMT, and *Akkermansia muciniphila* supplementation. **(A)** Representative immunofluorescence images of ectopic lesions from the EMs, EMs+Flu, EMs+ABX, and EMs+Flu+ABX groups. CD86 is shown in green, CD206 in magenta, and nuclei were stained with DAPI (blue). Scale bar: 50 μm. **(B)** Representative immunohistochemical images of CD86 and CD206 expression in ectopic lesions from the EMs, EMs+Flu, EMs+ABX, and EMs+Flu+ABX groups. Scale bar: 200 μm. **(C)** Representative immunofluorescence images of ectopic lesions from the EMs, EMs+Flu-FMT, EMs+EMs-FMT, and EMs+*A.muc* groups. CD86 is shown in green, CD206 in magenta, and nuclei were stained with DAPI (blue). Scale bar: 50 μm. **(D)** Representative immunohistochemical images of CD86 and CD206 expression in ectopic lesions from the EMs, EMs+Flu-FMT, EMs+EMs-FMT, and EMs+*A.muc* groups. Scale bar: 200 μm. *n* = 4 mice per group.

SUPPLEMENTARY TABLE 1 Differential taxa identified by Wilcoxon rank-sum test and LEfSe analysis in the metagenomic analysis.
