# Supplementary figures and images for: Gut microbiota-associated immunomodulation contributes to the protective effects of fluvastatin against endometriosis in a mouse model, accompanied by increased Akkermansia muciniphila abundance
Source: Front Microbiol. 2026 May 14;17:1762444. doi: 10.3389/fmicb.2026.1762444 (PMC13216716; doi:10.3389/fmicb.2026.1762444)

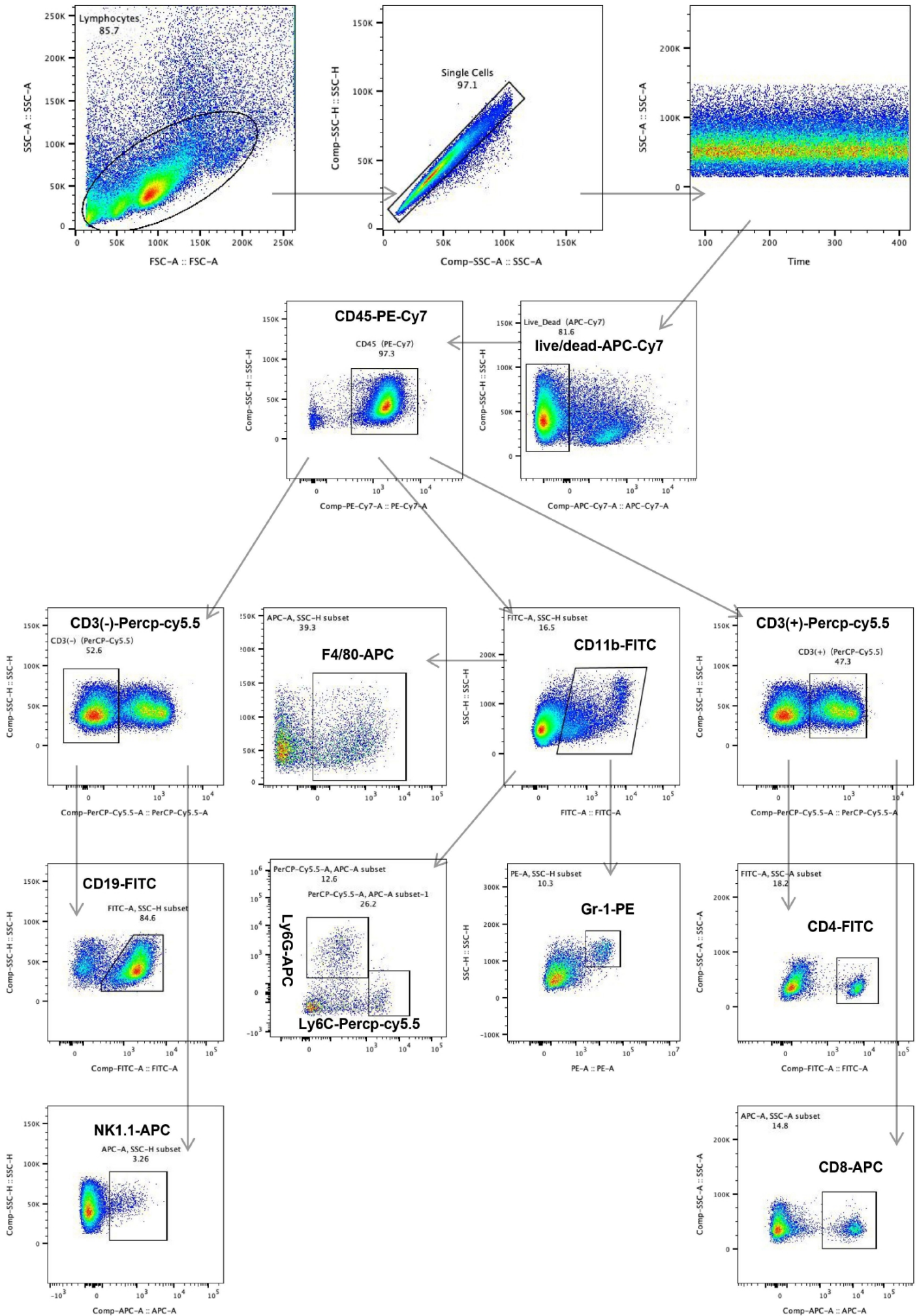

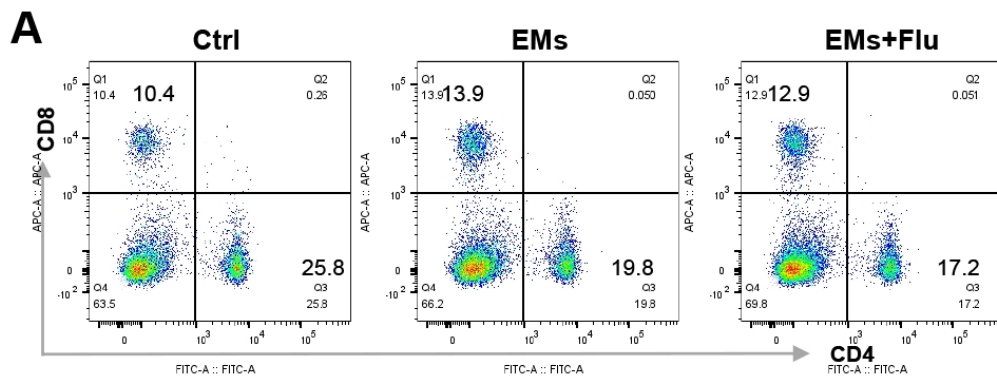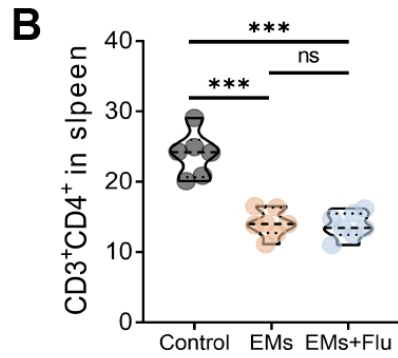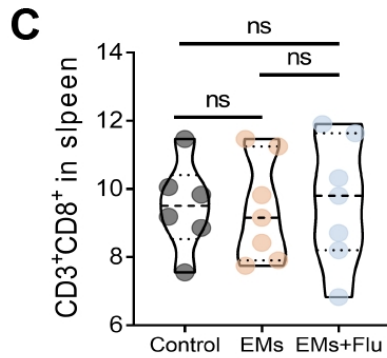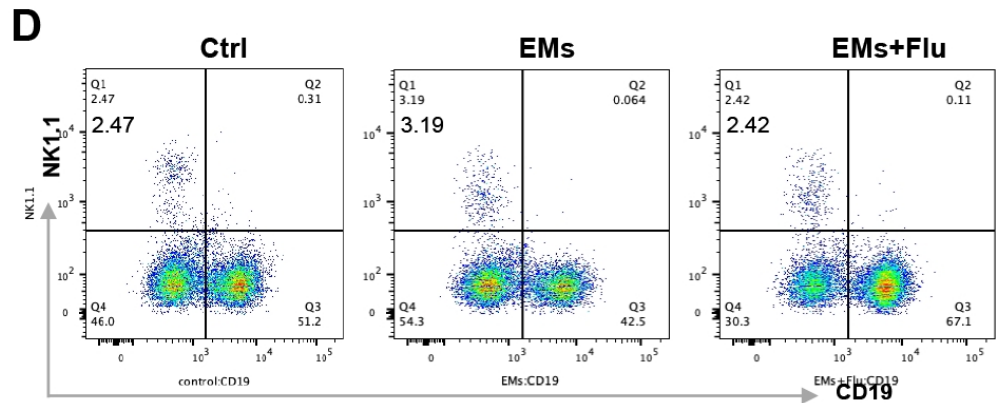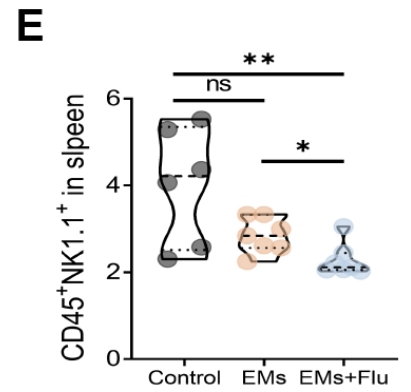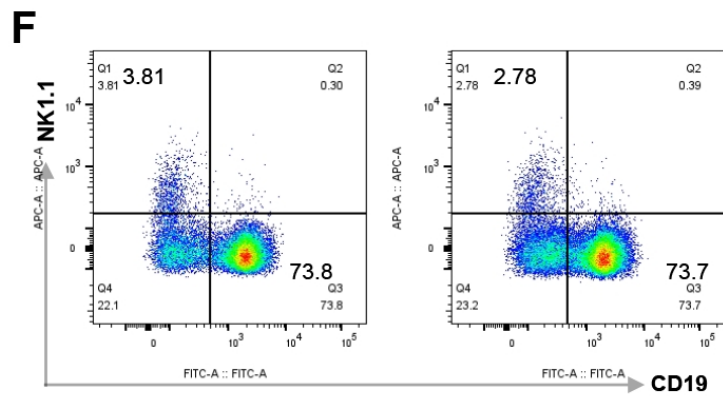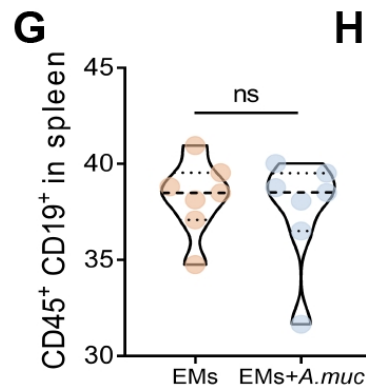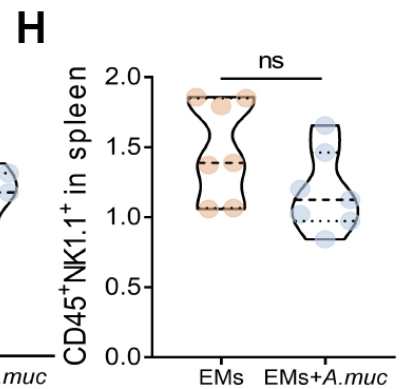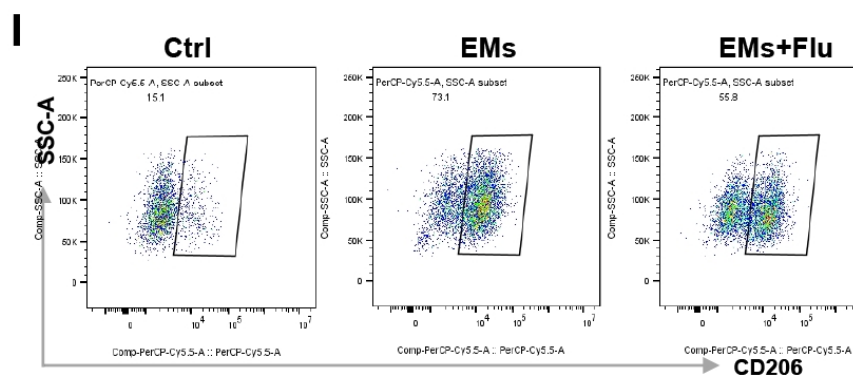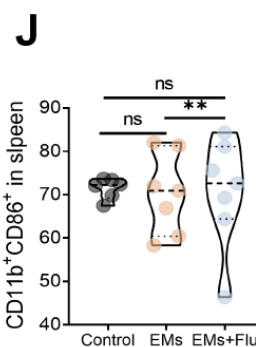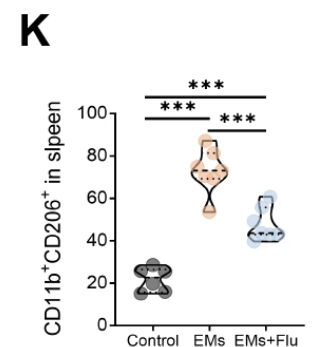

## A Total bacterial load

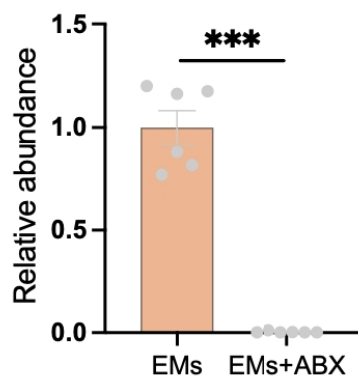

## B *A. muciniphila*

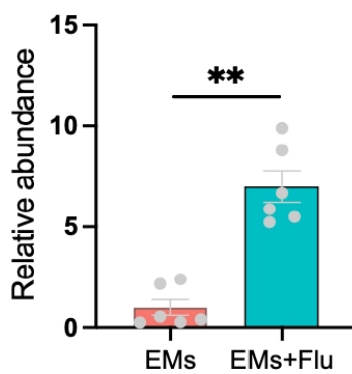

## *A. muciniphila*

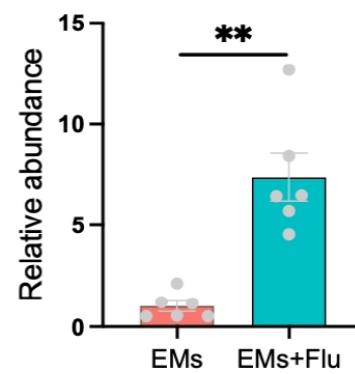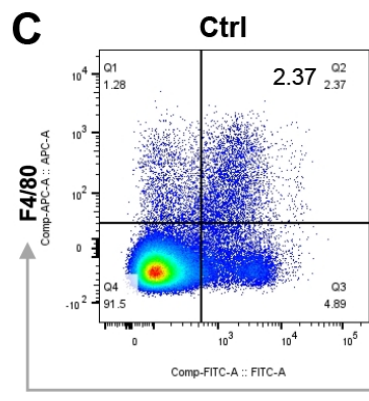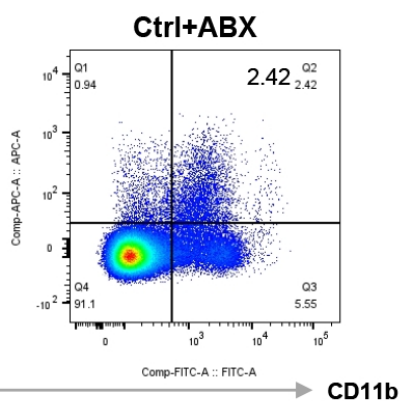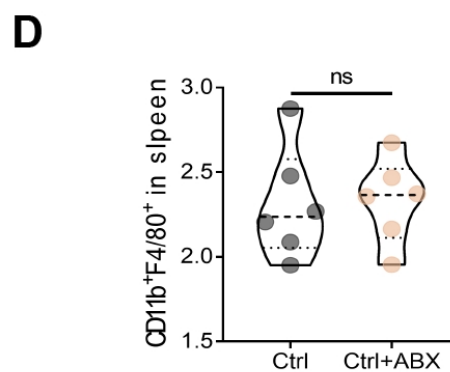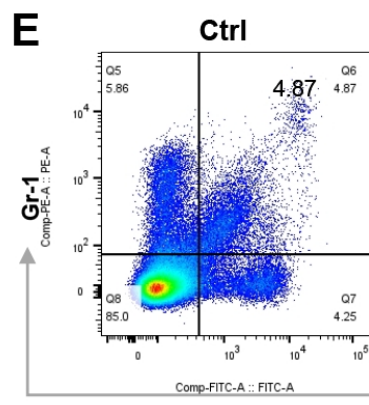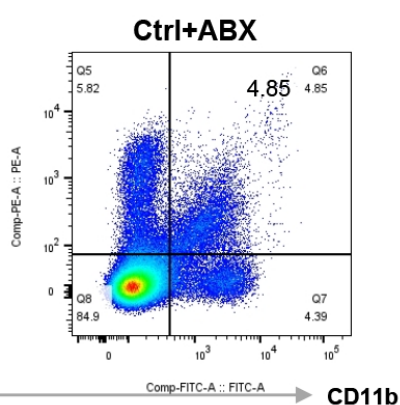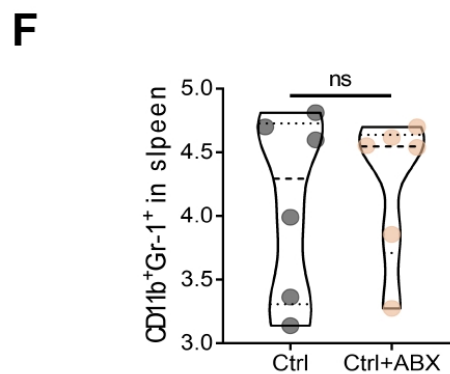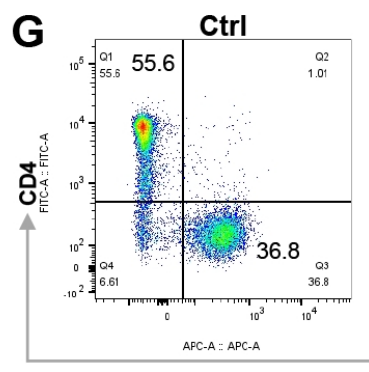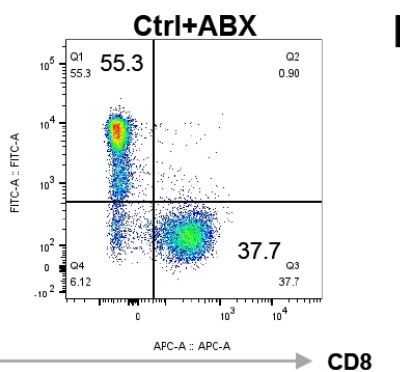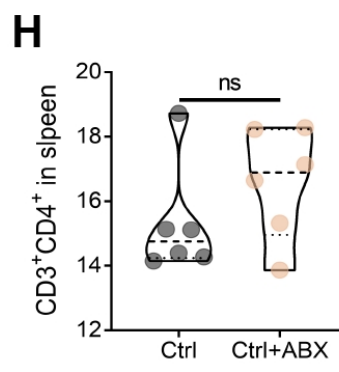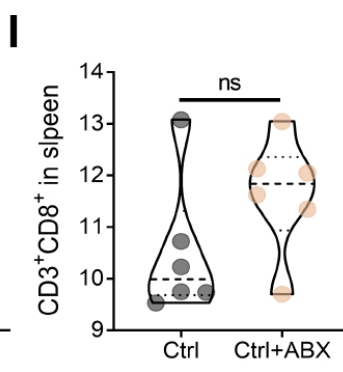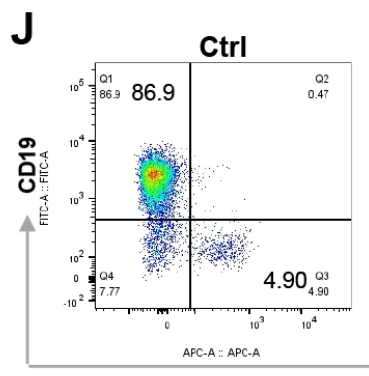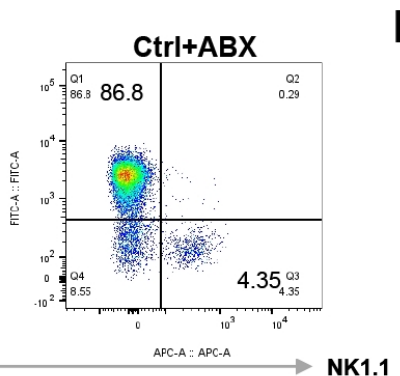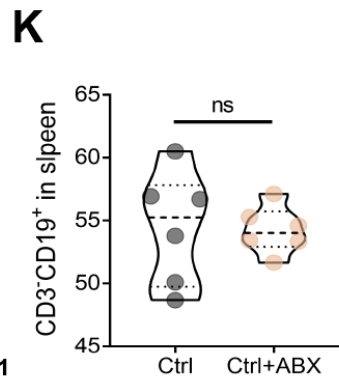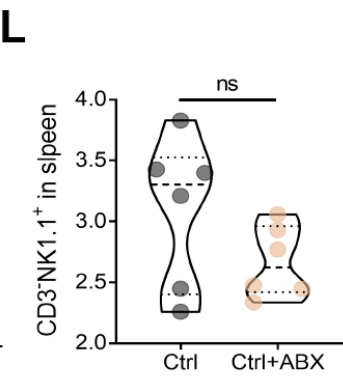

**A** CD86/CD206/DAPI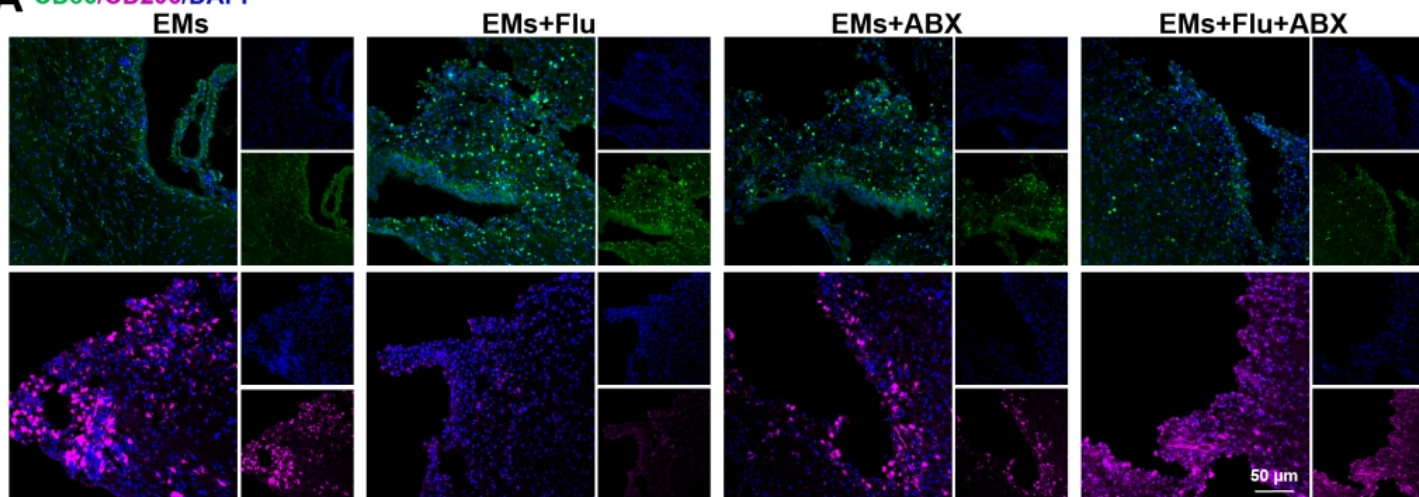**B**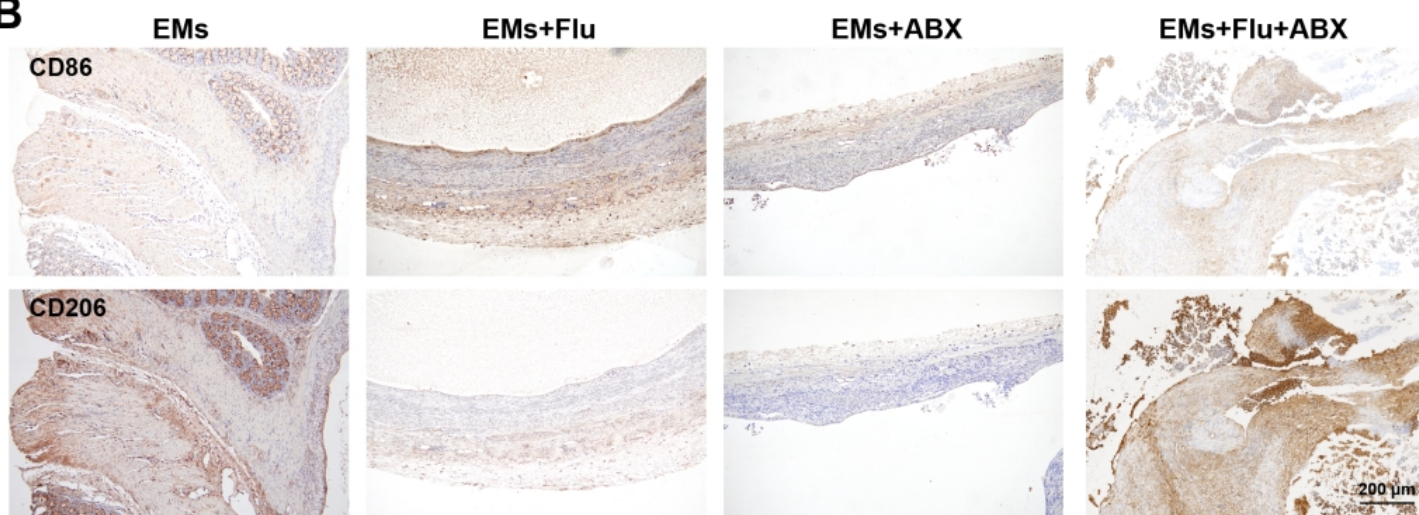**C** CD86/CD206/DAPI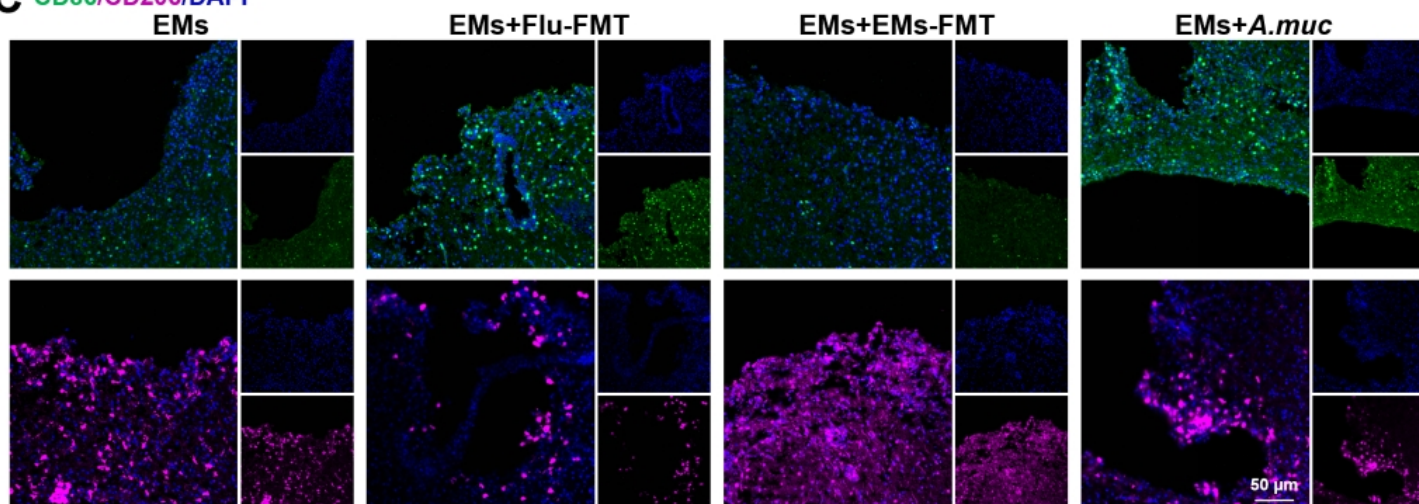**D**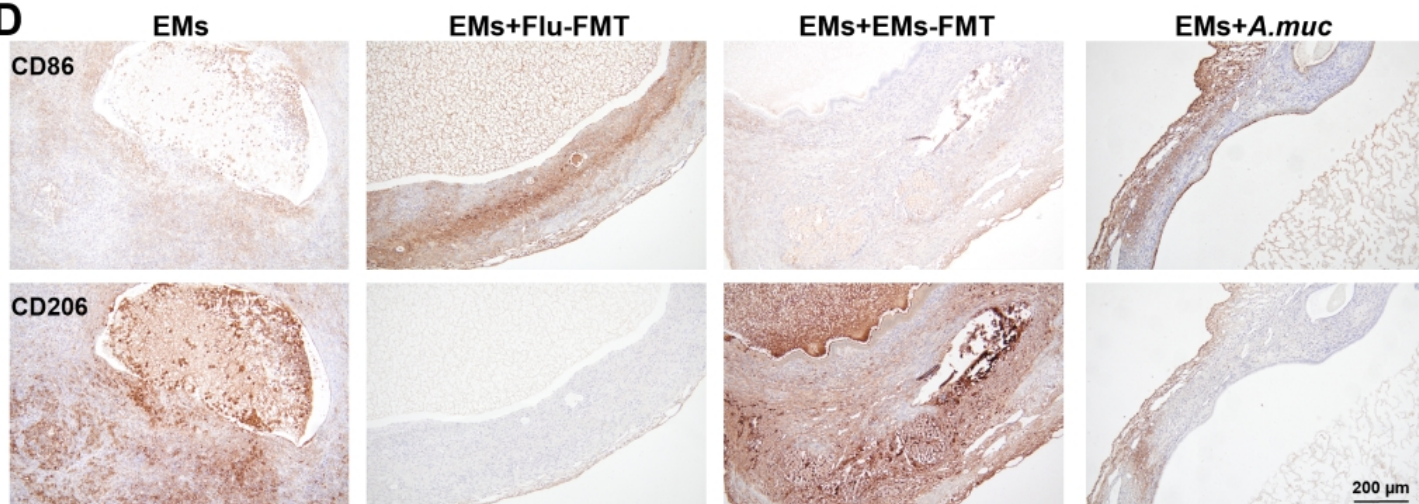

Supplement: SUPPLEMENTARY FIGURE S1 — Gating strategies used for flow cytometry analysis. [file Image_1.pdf]
